# Investigation of the Impact of a Protein Source on the Purification of l‑Asparaginase Type II from *Escherichia coli*

**DOI:** 10.1021/acsomega.5c01816

**Published:** 2025-06-13

**Authors:** Anna Catharinna da Costa, Talita Stelling de Araujo, Anna Carolina Lomba Pereira, Luis Ariel Espinosa Rodríguez, Leonardo Dingo do Lago, Camila Dias Leite da Silva, Rafael Alves de Andrade, Luís Maurício Trambaioli da Rocha e Lima, Fábio C. S. Nogueira, Gilberto Barbosa Domont, Marcius da Silva Almeida

**Affiliations:** † Protein Advanced Biochemistry (PAB), Institute of Medical Biochemistry (IBqM)—National Center for Structural Biology and Bioimaging (CENABIO), 28125 Universidade Federal do Rio de Janeiro, Rio de Janeiro RJ 21941-902, Brazil; ‡ Programa de Pós-Graduação em Química Biológica, Universidade Federal do Rio de Janeiro, Rio de Janeiro RJ 21941-902, Brazil; § Centro de Pesquisa em Medicina de Precisão (CPMP), Universidade Federal do Rio de Janeiro, Rio de Janeiro RJ 21941-902, Brazil; ∥ Laboratório de Biotecnologia Farmacêutica (pbiotech), Faculdade de Farmácia, Universidade Federal do Rio de Janeiro, Rio de Janeiro RJ 21941-902, Brazil; ⊥ Programa de Pós-Graduação em Ciências Farmacêuticas, Faculdade de Farmácia, Universidade Federal do Rio de Janeiro, Rio de Janeiro RJ 21941-902, Brazil

## Abstract

*Escherichia coli* l-asparaginase type II (EcA2) is essential for treating childhood acute lymphoblastic leukemia (ALL), improving survival rates since its introduction. After the expiration of its original patents, interest in producing biosimilars has increased, particularly to reduce treatment-related side effects. In this study, we compared two production methods for EcA2, purifying the enzyme from broth and from the soluble fraction of the cell pellet lysate. Using a converging purification workflow, we obtained 66.3 (±2.3) mg/L of l-asparaginase from broth and 29.2 (±4.6) mg/L from the cell pellet lysate, with specific activities of 136.3 (±13.3) and 123.5 (±10.3) IU/mg, respectively. Both versions showed similar three-dimensional structures, thermal stability, and specific activity, with no significant differences in performance. Proteomic analysis revealed that purification from broth resulted in fewer host cell proteins than purification from the cell pellet lysate. Our results further suggest that the purification process from cell lysate is more susceptible to variability than purification from the broth. These findings demonstrate that while both production methods yield comparable enzymes in terms of structure and activity, purifying from broth may offer advantages in terms of lower contamination and better reproducibility.

## Graphical abstract

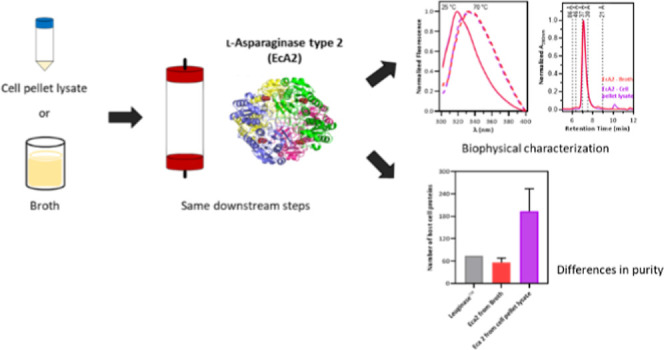

## Introduction

The enzyme l-asparaginase type II from *Escherichia coli* is fundamental in the chemotherapy treatment of childhood acute lymphoblastic leukemia (ALL), the most prevalent pediatric cancer.^1–6^ Since its insertion in the therapeutic protocol, it has been shown to significantly improve the survival rate by around 30% to 80% in pediatric patients.^7,8^ Its mechanism of action involves the hydrolysis of l-asparagine in l-aspartate and ammonia.^9^ The resulting depletion of serum l-asparagine leads to the death of tumor cells, which require this amino acid from the serum for their survival, due to nonsignificant expression of the enzyme asparagine synthetase.^10,11^

The interest in the production of l-asparaginases with antitumor activity started in the 1950s, after the initial observation by Kidd that mice with lymphomas treated with guinea pig serum (*Cavia porcellus*) showed rapid regression of the disease.^12,13^ A few years later, the antitumor properties of *C. porcellus* serum were attributed to the enzymatic activity of an l-asparaginase,^14^ and since then, research has focused on different sources of this enzyme to enable its large-scale production. In 1964, Mashburn and collaborators reported that l-asparaginase from *E. coli* has antitumor growth activity very similar to that of *C. porcellus* serum.^15^ These findings served as the basis for the purification,^16,17^ followed by the large-scale production of this enzyme from bacteria, and for conducting preclinical and clinical studies.^18^ This culminated in the approval by the FDA, in 1978, of the first formulation containing l-asparaginase type II from *E. coli*, marketed under the name of Elspar (Merck, West Point, PA, USA).

After the expiration of the first patent protecting l-asparaginase type II in the 21st century, there has been increasing interest in the production of biosimilars to this enzyme. This shift in focus can be measured by the number of scientific manuscripts published annually on the topic, which ranged between 0 and 1 from the 1970s until steadily increasing in the 21st century, peaking at thirty-one manuscripts in 2023 (Scopus search performed in May 2024 using keywords “l-asparaginase” AND “leukemia” AND “production” within the title, abstract, and keywords). This was followed by a change in manufacturers of this active pharmaceutical ingredient, ultimately leading to significant price fluctuations, shortages, and the introduction of substandard preparations of this essential medicine.^19–21^

Even though many species of bacteria, fungi, plants, and animals produce l-asparaginases, only the enzymes derived from *E. coli* and *Erwinia chrysanthemi* are used in the treatment of onco-hematological malignancies, mainly in the treatment of acute lymphoblastic leukemia.^22^

The therapeutically relevant l-asparaginases are usually produced recombinantly, as the extraction of enzymes directly from native organisms results in lower yields compared to recombinant production. Numerous protocols are available in the literature.^23–27^ These protocols include various plasmid constructs that lead to intracellular or periplasmic l-asparaginase expression. It is worth noting that many recombinant proteins, including l-asparaginase, are often transferred from the periplasm to the broth. The source of the protein, whether from the cell mass or the broth, is particularly important for downstream processing at an industrial scale, where production costs can vary significantly depending on the methods used.^28–32^

Despite the technological importance of the source of l-asparaginase type II, whether intracellular or extracellular, we did not find any studies that experimentally compared these two production protocols in a way that would allow for a rational decision on the method of recombinant expression of this enzyme in *E. coli*. In fact, the literature includes cases of optimization for protein purification from cell lysate as well as cases where the protein is captured from the broth, discarding the cell mass.

In this work, we report a comparability characterization of the purity, structure, and activity of recombinant l-asparaginase type II purified from either *E. coli* lysates or broth.

## Results

### Workflow for Investigating the Influence of a Protein Source on the Purification of *E. coli* l-Asparaginase Type 2

In this study, two recombinant production processes for l-asparaginase type 2 from *E. coli* (EcA2) were conducted to investigate the impact of the protein source on the purification steps, as well as on the three-dimensional structure, specific activity, and purity of the enzymes at the end of the process (Figure 1). As shown in the workflow, both enzymes were purified using the same chromatographic methods (hydrophobic interaction and ion exchange) and monitored by denaturing polyacrylamide gel electrophoresis. In the end, the 3D structure of purified EcA2 was evaluated by intrinsic fluorescence, nuclear magnetic resonance, and analytical gel filtration. Mass spectrometry experiments were conducted to evaluate the chemical integrity of the proteins produced as well as their purity by identifying residual host cell proteins (HCPs). Furthermore, specific activity was analyzed by direct absorptiometry of l-asparagine.

**Figure 1:**
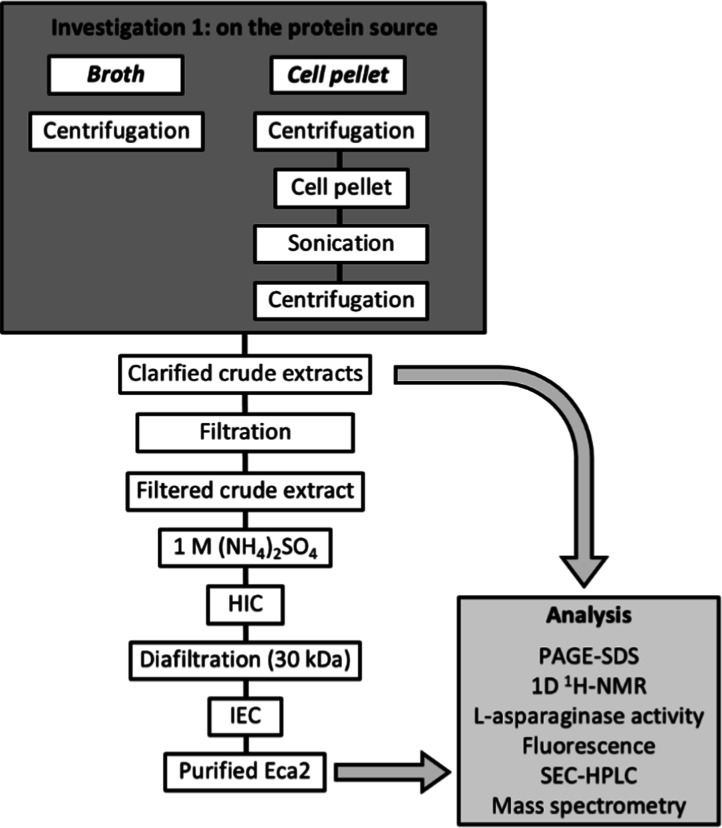
Workflow for investigating the influence of the protein source on the purification of l-asparaginase type 2 from *E. coli*. The purification steps and the analytical methods are indicated for each designed protocol.

### Recombinant l-asparaginase Expression

Two culture media were used to produce l-asparaginase in shake flasks: LB medium, to produce the protein intracellularly (in the cell pellet), and TB medium, to produce the protein in the culture supernatant. The *E. coli* BL21 (DE3) strain was used for recombinant enzyme production. The recombinant protein expression was induced with 0.2 mM IPTG at 37 °C and 200 rpm, as described in the Materials and Methods section.

Since the plasmid contains the pelB export sequence, which targets the recombinant protein to the periplasm, it can be observed in the 15% SDS-PAGE (Figure 2) that the protein leaked into the culture medium after expression, regardless of the cultivation method. However, as shown in Figure 2A, l-asparaginase expressed in TB medium is predominantly found in the broth supernatant, while in LB medium, l-asparaginase is primarily located in the cell pellet (Figure 2B).

**Figure 2:**
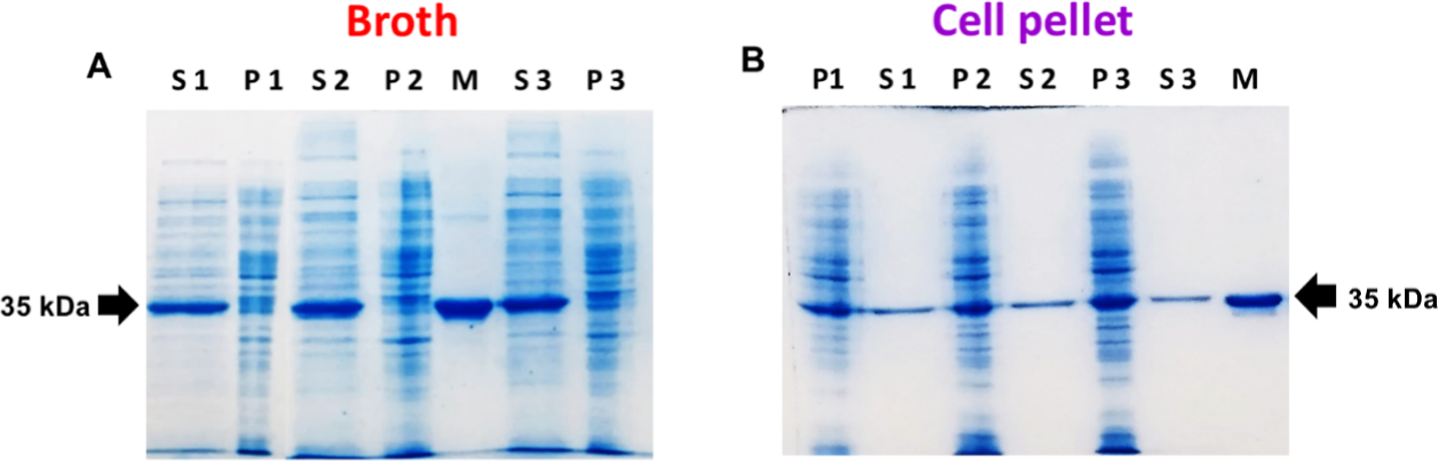
Expression of l-asparaginase type 2 from *E. coli* BL21 (DE3) in (A) supernatant using terrific broth and (B) cell pellet using Luria–Bertani broth. Protein content was visualized by 15% SDS-PAGE stained with Colloidal Coomassie Blue. Labels: M, EcA2 used as a molecular mass standard (35 kDa); S1, S2, and S3: replicates of culture supernatants at the end of recombinant expression; P1, P2, and P3: replicates of soluble fractions from cell pellet lysates at the end of recombinant expression (uncropped gel in the Supporting Information, Figure S1).

### Investigation of Protein Capture Methods

As seen in Figure 3A (HIC from broth) and 3B (HIC from the soluble fraction of cell pellet lysate), l-asparaginase was eluted in fractions 39–49, as indicated by 15% SDS-PAGE. The chromatogram corresponding to the HIC of the soluble fraction of cell pellet lysate shows a higher amount of bound material compared to the HIC of the broth, likely due to the greater number of contaminating proteins from the cell pellet lysis, which were detected by 15% SDS-PAGE (uncropped gels in the Supporting Information, Figure S3).

**Figure 3:**
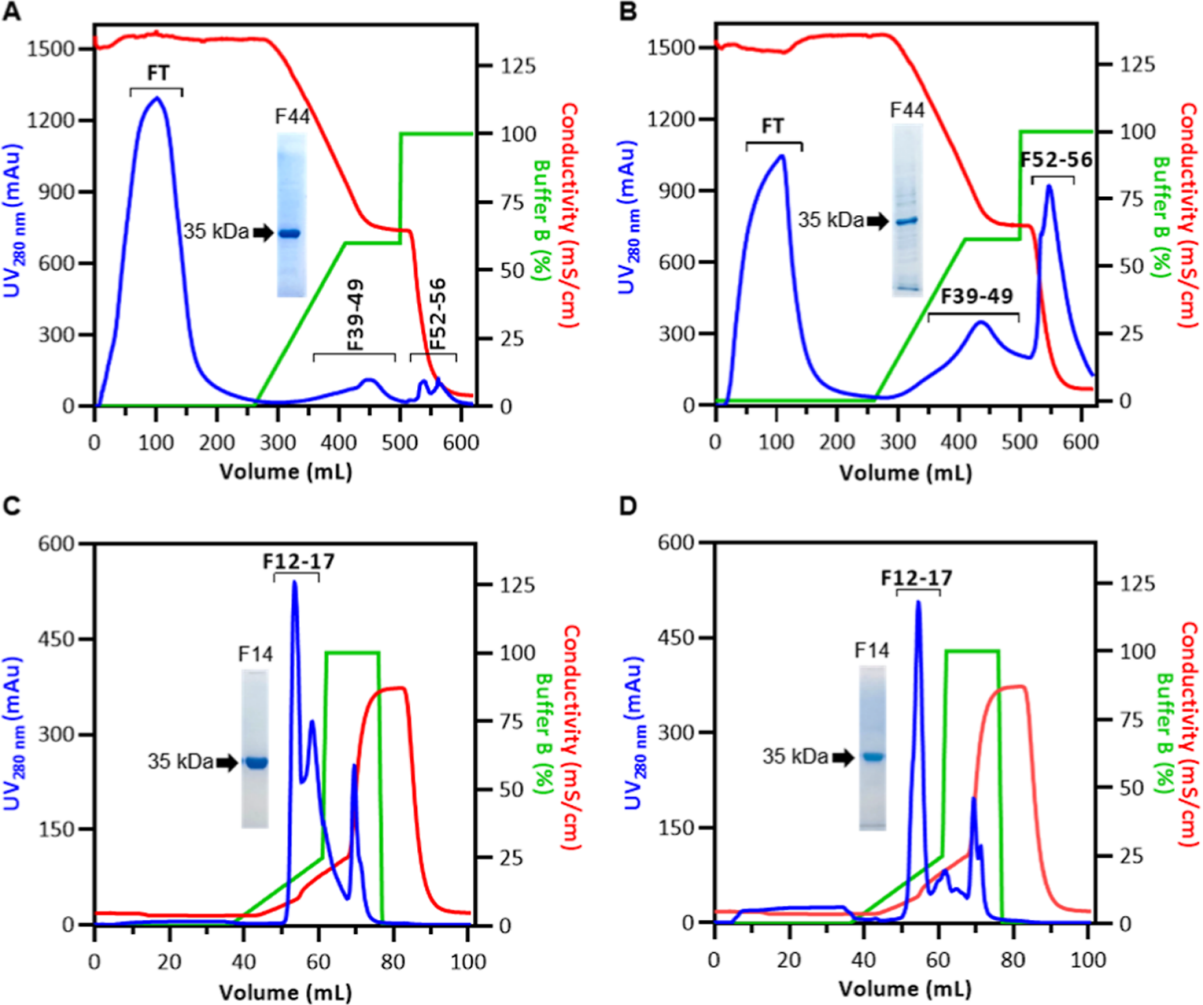
Chromatographic profiles of EcA2 purification. Hydrophobic interaction chromatography profiles for EcA2 capture from broth (A) or soluble fraction of cell pellet lysate (B). Anion exchange chromatography profiles following hydrophobic interaction chromatography from broth (C) or soluble fraction of cell pellet lysate (D). The blue lines in the chromatograms indicate UV absorption monitoring at 280 nm, the red lines indicate conductivity, and the green lines represent the percentage of buffer B. Fractions from each chromatography were evaluated by 15% SDS-PAGE stained with colloidal Coomassie Blue, and the fraction corresponding to the middle of the peak containing EcA2 is shown in each chromatogram.

In the ion exchange chromatography (Figure 3C—IEC from broth and 3D—IEC from the soluble fraction of cell pellet lysate), l-asparaginase was eluted in fractions 12–17, as indicated by 15% SDS-PAGE. After this second chromatography step, no protein contaminants were visible in the 15% SDS-PAGE for either the enzyme purified from the broth or in the soluble fraction of the cell pellet lysate (uncropped gels in the Supporting Information, Figure S3).

### Enzymatic Activity

The specific activity of l-asparaginase purified from the broth or the soluble fraction of the cell pellet lysate was evaluated by monitoring the hydrolysis of l-asparagine. As shown in Figure 4, the specific activities of the enzymes are comparable, with EcA2 purified from the soluble fraction of the cell pellet lysate exhibiting slightly lower specific activity. However, an unpaired *t*-test with Welch’s correction (parametric) indicated no statistical difference between the specific activity of these samples (*p* = 0.49). A *p* value of <0.05 was established as the criterion for statistical significance.

**Figure 4:**
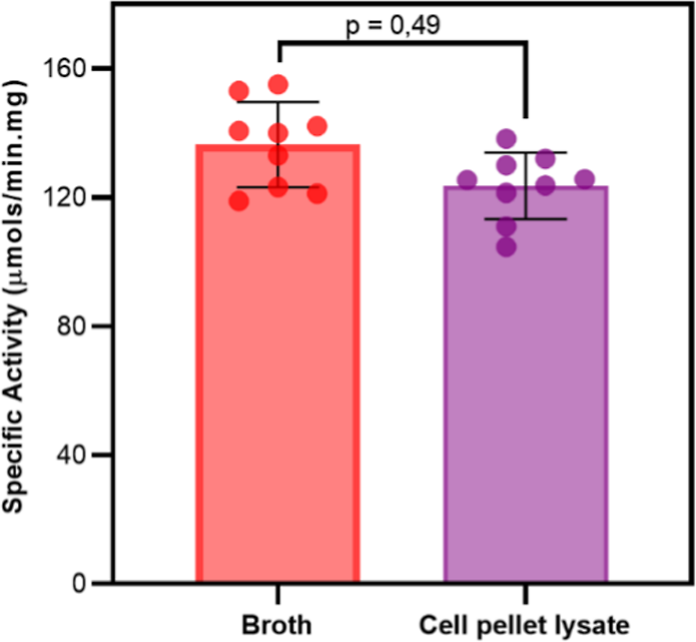
Specific activity of l-asparaginase purified from broth (red) or the soluble fraction of the cell pellet lysate (purple). The hydrolysis of 20 mM l-asparagine was monitored by absorption at 225 nm in 100 mM sodium phosphate, pH 8.0, with 50 μg/mL of each enzyme at 37 °C. For statistical evaluation, an unpaired *t*-test with Welch’s correction was applied, assuming a Gaussian distribution of the data. Experiments were performed in triplicate with three independently purified samples.

### Biophysical Characterization of l-Asparaginase Type 2

1D (^1^H)-NMR experiments were conducted to evaluate the three-dimensional structure of l-asparaginase purified from the broth or the soluble fraction of the cell pellet (Figure 5A). The spectra display well-dispersed signals over a wide range of chemical shifts (from 0.5 to 10 ppm), characteristic of proteins with a well-defined three-dimensional structure. Additionally, most of the peaks are broad, which is the expected profile for proteins with high molecular weights (approximately 141 kDa for the EcA2 tetramer).

**Figure 5:**
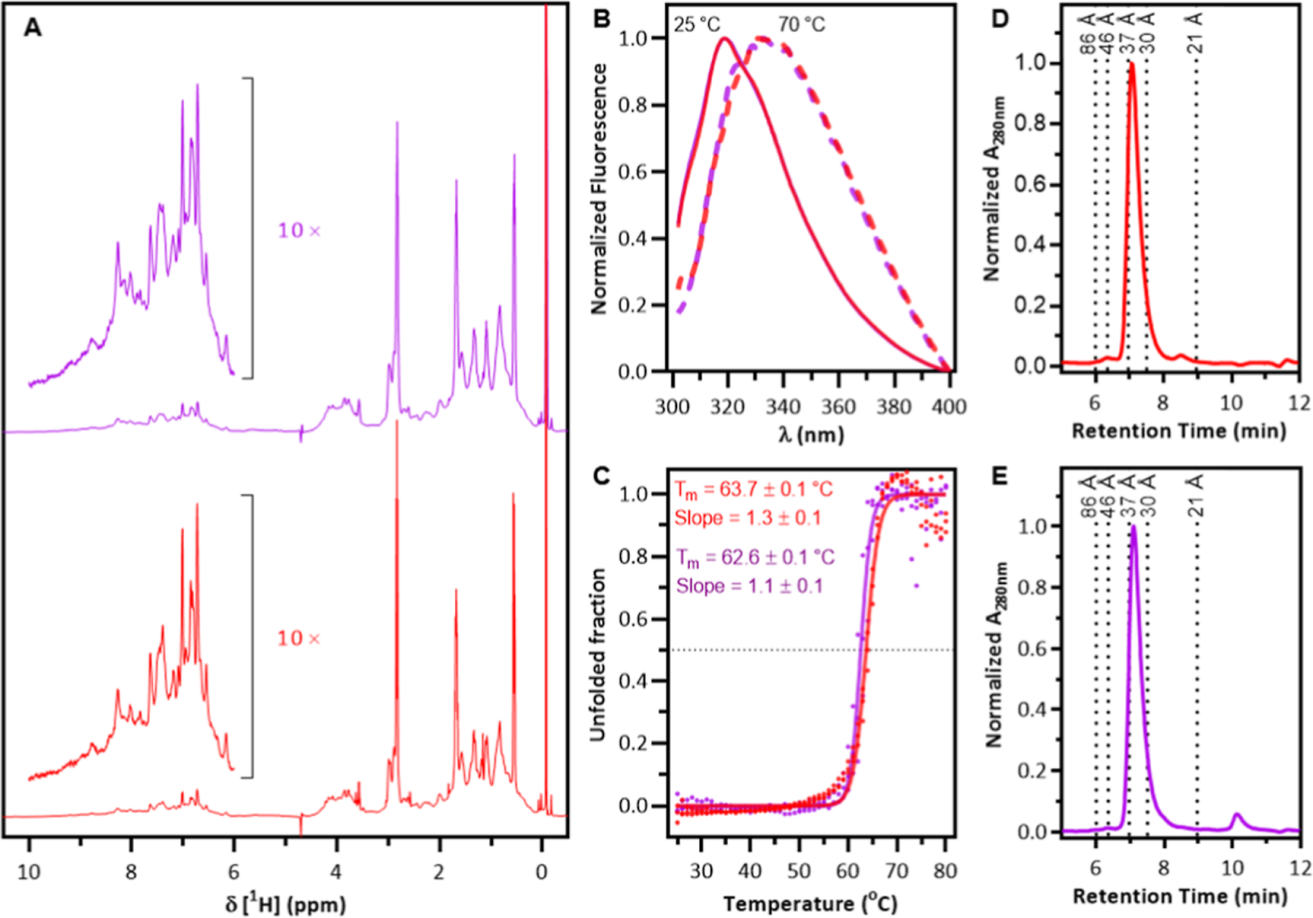
Biophysical analysis of l-asparaginase type 2 purified from broth (red) or soluble fraction of cell pellet lysate (purple). (A) 1D ^1^H-RMN spectra were collected at 25 °C on a Bruker Avance III spectrometer operating at a ^1^H frequency of 600 MHz. The ^1^H chemical shift was referenced to 3-(trimethylsilyl)-1-propanesulfonic acid, sodium salt (strong peak at 0 ppm). Insets present a region of the spectra where the intensity scale was increased 10× for clarity. (B) Fluorescence emission spectra of enzymes in 25 mM sodium phosphate buffer, pH 7.4. Solid lines represent spectra collected at 25 °C, and dashed lines indicate spectra collected at 70 °C. Intrinsic fluorescence spectra were collected at an excitation wavelength of 295 nm (see Supporting Information, Figure S4). (C) Thermal denaturation curves monitored by intrinsic fluorescence. The experimental data was fitted to the Boltzmann sigmoidal equation, using the GraphPad Prism software (version 8.0.2), to identify the melting temperature (*T*_m_) of the proteins. (D,E) Analytical size exclusion chromatography profiles of l-asparaginase purified from broth (red) or soluble fraction of cell pellet lysate (purple). The dotted lines indicate the retention times of the globular proteins used as standards: 86 Å (thyroglobulin), bovine serum albumin (46 Å dimer and 37 Å monomer), 30 Å (ovalbumin), 21 Å (standard curve in the Supporting Information, Figure S6). OD_280nm_ was normalized to facilitate comparison of chromatograms. Experiments were performed in triplicate.

To investigate the influence of the protein source on global conformation and thermal stability, a thermal denaturation experiment was performed, monitoring the intrinsic fluorescence emission spectra (Figure 5B,C). At 25 °C, the maximum fluorescence emission for both enzymes occurred at 320 nm, indicating that they were well-folded. As the temperature increased, the maximum fluorescence emission shifted to 340 nm, a strong indication that the enzymes had denatured. Two well-defined conformational states are evident (Figure 5C), with abrupt pre- and post-transition phases, suggesting cooperativity in the process. Furthermore, no significant differences in the transition temperature (*T*_m_) or slope were observed between the enzymes.

Analytical size exclusion chromatography was performed using a TSK-Gel G2000SW_XL_ column to characterize the oligomeric distribution of the type 2 l-asparaginase enzyme purified from the broth or the soluble fraction of the cell pellet lysate (Figure 5D,E). The enzymes exhibited a very similar oligomeric distribution, with comparable elution times at 7.1 min, which correspond to a protein with 36.2 Å of hydrodynamic radius. These results indicate that the protein source, whether from broth or cell pellet, has no major impact on the conformation in solution or thermal stability.

### Analysis of Homogeneity and Identity of l-Asparaginase Type 2 Samples Purified from Broth or Cell Pellet Lysate

After the purification protocol, aliquots of l-asparaginase from each triplicate were combined to assess the homogeneity of the enzymes and their identity after expression from two different sources. A serial dilution of the l-asparaginase samples was performed, and as shown in the 15% SDS-PAGE gel in Figure 6, both the enzyme purified from the broth and the soluble fraction of the cell pellet lysate displayed an electrophoretic mobility profile equivalent to a biosimilar product. However, another protein band was detected in the purified sample from the soluble fraction of the cell pellet lysate.

**Figure 6:**
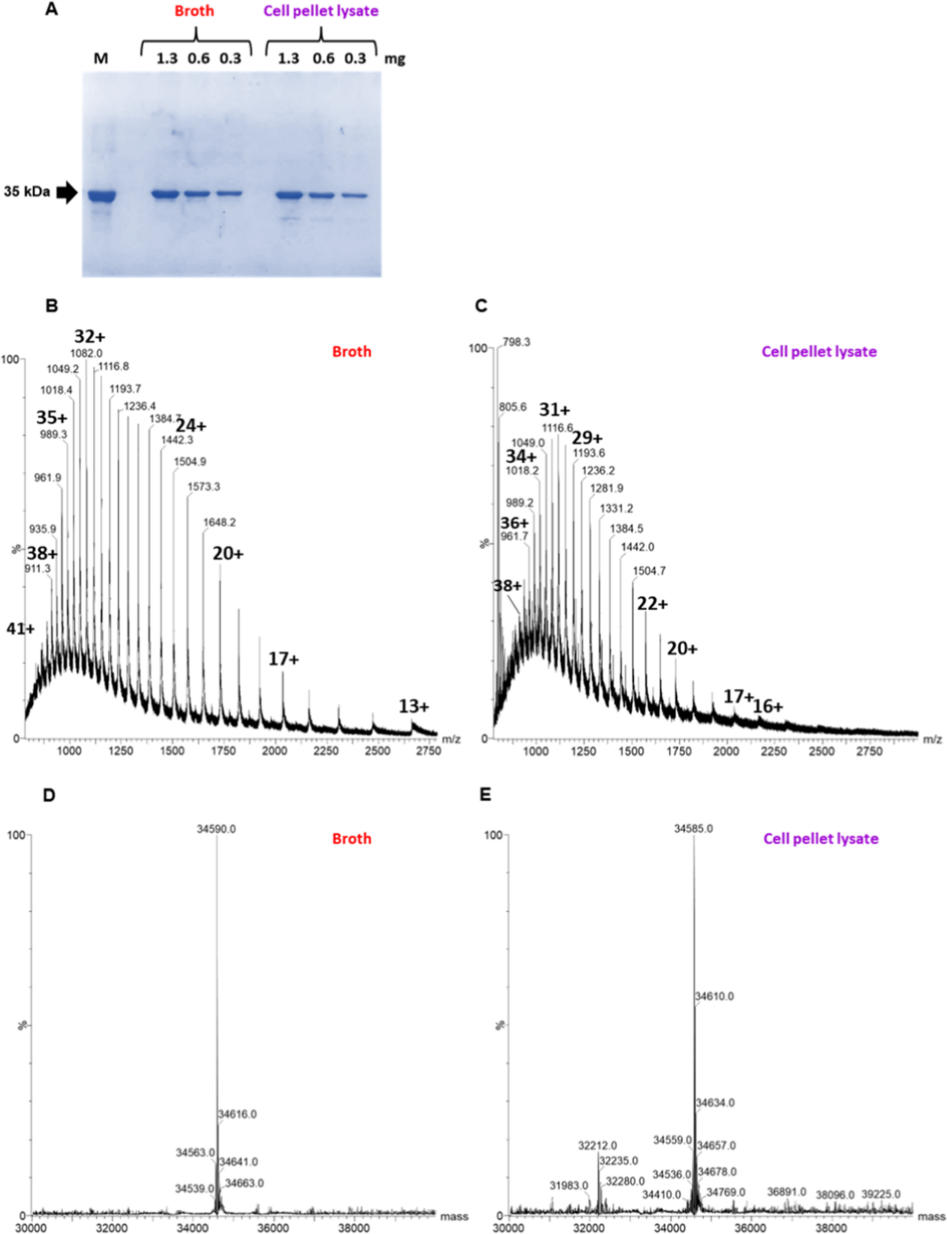
Homogeneity and identity of l-asparaginase type 2 samples purified from broth or cell pellet. (A) SDS-PAGE gel stained with colloidal Coomassie Blue. Lanes: M, EcA2 used as molecular mass standard (35 kDa); broth: l-asparaginase type 2 purified from the culture supernatant after the hydrophobic interaction and ion exchange chromatography; cell pellet: l-asparaginase type 2 expressed purified from the cell pellet after the hydrophobic interaction and ion exchange chromatography. The amount of protein loaded in each lane is indicated. (B–E) Characterization by ESI–MS. Charge envelopes (B,C) and deconvoluted spectra (D,E) are presented for purified EcA2 from broth or cell pellet.

The intact products of l-asparaginase type 2 were evaluated by ESI–MS, revealing a major peak in the mass spectra of each sample (Figure 6B–E). The deconvoluted spectra indicated a mass of 34 590 Da for the enzyme purified from the broth (Figure 6D) and 34 585 Da for the enzyme purified from the soluble fraction of the cell pellet lysate (Figure 6E). The sample from the soluble fraction also contained additional species with significant peak intensities, indicating the presence of contaminants. Furthermore, the MS spectra of l-asparaginase purified from the cell pellet revealed a 798 *m*/*z* ion with a +3 charge (Figure 6C), consistent with a peptide fragment having a monoisotopic mass of 2931 Da—equivalent to a peptide originating from the first 25 amino acids of the mature EcA2 sequence. Additionally, the spectra in Figure 6D and 6E identified adducts of +26 Da related to acetaldehyde, which was confirmed as one of the eight most abundant modifications detected in the HCP profile (see Figure S8), primarily in the cell pellet lysate and Leuginase samples. Reference 33 reported this same modification in several lysine residues of carbonic anhydrase II (CA II) by ESI–MS analysis (ESI FT-ICR).

### Host Cell Protein Analysis by LC–MS/MS

LC–MS/MS analysis and protein identification were performed on three samples purified from the broth (S1–S3), on three samples from the soluble fraction of the cell pellet lysate (P1–P3), and on a commercial comparator protein from Leuginase (Leu; see Supporting Information, Table S1). The reference proteome of the BL21­(DE3) strain (UP000002032_469008) was used for the identification. An initial search using the *FragPipe* platform identified EcA2, along with a total of 352 host cell-contaminating proteins (HCPs), each having at least 2 unique spectral counts and 2 unique peptides. A summary of the number of identified proteins, peptides, peptide spectral matches (PSMs), total spectral counts (TSC), HCP spectral counts, and percentage of EcA2 or HCP (based on TSC) is shown in Figure 7A–C.

**Figure 7:**
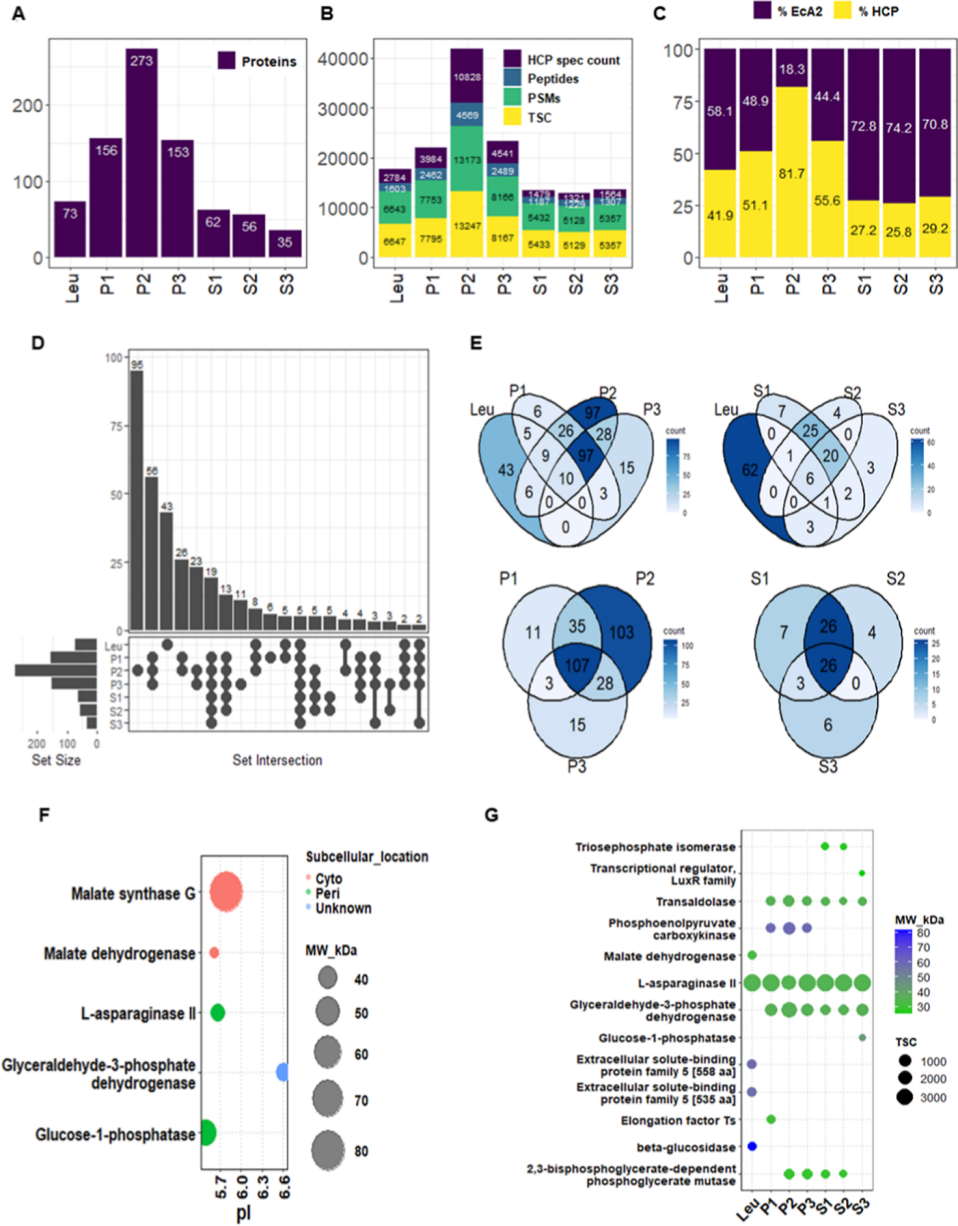
Broth samples showed a lower number of proteins and spectral count of HCP when compared to those from the cell pellet. (A) Summary of the LC–MS/MS analysis with the number of identified proteins; (B) PSMs (peptide spectral matches, in green), total spectral count (in yellow), peptides (in blue), and HCP spectral count (in purple) in each sample analyzed; (C) relative abundance of total HCPs; (D) upset plot comparing the identified proteins across all samples; (E) Venn diagrams of count identified proteins in Leuginase vs the samples purified from the soluble fraction of the cell pellet lysate, Leuginase vs the samples purified from the broth, and within samples purified from the soluble fraction of the cell pellet lysate and from the broth; (F) dot plot of the 5 most common proteins across all samples showing the subcellular location (Cyto: cytoplasm, Peri: periplasm), molecular weight (MW), and isoelectric point (pI); (G) dot plot of the most abundant proteins, based on total spectral counts (TSC—circle sizes) in each sample, with color indication of their molecular weight.

The samples purified from the broth yielded the lowest number of proteins (S3 < S2 < S1, ranging from 35 to 62 proteins; see Figure 7A), followed by Leuginase with 73 proteins. In contrast, the samples purified from the soluble fraction of the cell pellet lysate had the highest protein counts (P3 < P1 < P2, ranging from 153 to 273 proteins). EcA2 (A0A140N7Y9) was the most abundant protein, with the highest peptide spectral count among all identified proteins, except in the P2 sample, where glyceraldehyde-3-phosphate dehydrogenase (A0A140N783) had the highest peptide spectral count (Table S1). Figure 7C shows that the relative abundance of EcA2 (based on total spectral count) in the broth was higher (70–74%) compared to that of Leuginase (58%) and the cell pellet lysate samples (18–48%). The relative abundance of total host cell proteins (HCPs) (Figure 7C) ranged from 25–29% in the broth samples (S1 to S3), 42% in Leuginase, and 51–81% in the samples purified from the cell pellet lysate (P1 to P3), with P2 being an outlier at 81%.

An upset plot comparing the seven samples (Figure 7D) reveals that sample P2 contains 95 unique proteins and that 56 proteins are shared among the three biological replicates of the cell pellet lysate (P1–P3). In addition, Leuginase contained 43 proteins that were exclusive to it, while five proteins were common to all seven samples. Subsequently, we compared Leuginase to both the cell pellet lysate and the broth. The results (Figure 7E) indicate that ten proteins are common between Leuginase and the cell pellet lysate and six proteins are common between Leuginase and the broth. Moreover, 62 proteins were detected exclusively in Leuginase when compared to the broth samples (Leu vs broth 1–3). Within the cell pellet lysate (Figure 7E, cell pellet lysate 1–3), an analysis identified 107 proteins common to all three replicates, with P2 containing the highest number of unique proteins (103), compared to 15 in P3 and 11 in P1. Similarly, in the samples purified from the broth (Figure 7E, broth 1–3), 26 proteins were common, with each sample having a low number of unique proteins (ranging from 4 to 7).

A detailed analysis of the five proteins common to all samples (Figure 7D) identified EcA2 and four host cell proteins (HCPs) corresponding to malate synthase G, malate dehydrogenase, glyceraldehyde-3-phosphate dehydrogenase (G3PDH), and glucose-1-phosphatase (G1P) (Figure 7F). All of these proteins have similar molecular weights, ranging from 32 to 43 kDa, except for malate synthase G, which has a molecular weight of 80 kDa. The isoelectric points (pI) of the four proteins ranged from 5.49 to 5.79, whereas G3PDH had a pI of 6.61. Furthermore, among the HCPs, two proteins were localized in the periplasm and two in the cytoplasm. A complete analysis of protein molecular weight (kDa) versus pI (and including total spectral count, TSC) for each sample (Figure S7) shows that the broth samples exhibit a narrow distribution, while the pellet samples display a broader distribution. In contrast, Leuginase has a narrow range of pI values and a wider range of molecular masses.

An analysis of the top five most abundant proteins (Figure 7G), based on spectral counts in each sample, revealed two common proteins between the broth and pellet fractions: G3PDH and transaldolase, which have molecular weights similar to that of EcA2. Additionally, 2,3-bisphosphoglycerate-dependent phosphoglycerate mutase was detected in samples purified from both the broth and the soluble fraction of the cell pellet, although it was absent in the P1 and S3 samples. G3PDH was the most abundant host cell protein in the P2 sample, even exceeding that of EcA2 in abundance. In our study, the HCP profile among the top five most abundant proteins in Leuginase differed from that observed in the samples purified from the broth and the soluble fraction of the cell pellet lysate but was consistent with the findings reported by ref 34.

An open search in *FragPipe* using the *PTM-Shepperd* module was performed to explore possible modifications that were not detected in the closed search. Figure S8 shows the eight most abundant modifications—each representing 1–3.8% of the total PSMs—distributed across all samples. Deamidation, carbamylation, and the formation of pyroglutamic acid from E (−H_2_O) and Q (−NH_3_) were the four most enriched modifications. Carbamylation is introduced by the use of urea in the experimental protocol, while the remaining modifications may result from storage conditions, pH, temperature, and purification procedures. Formylation (+28 Da) and acetaldehyde (+26 Da) modifications were also detected; interestingly, acetaldehyde modification was highly enriched in the Leuginase and cell pellet lysate samples. This modification could explain some of the adducts detected at the protein level in both samples.

The EcA2 sequence (L23–Y348) from K12 and BL21 (DE3) strains exhibits four amino acid variations at positions 49 (V/A), 86 (N/D), 274 (S/T), and 285 (T/N) (see Figure S9 for sequence differences). To account for these differences, we performed a second closed search in the *MSFragger* engine using both EcA2 sequences—each with and without the N-terminal signaling peptide (1MKYLLPTAAAGLLLLAAQPAMA22_Q04085_pelB or 1MEFFKKTALAALVMGFSGAALA22_A0A140N7Y9). Neither the native signaling peptide (A0A140N7Y9) nor the plasmid-derived signal peptide (pelB) was detected in the LC–MS/MS analysis. MS/MS spectra (Figure S11) confirmed that the N-terminal sequence of EcA2 spans L23–K51 and verified the substitution at position 49 in the BL21 variant (i.e., the presence of alanine). The N-terminus of EcA2 detected in samples purified from both the broth and the soluble fraction of the cell pellet lysate is consistent with the findings for Aginasa.^34^ Finally, the C-terminal end was confirmed—despite the absence of a basic residue—by identifying sequence 337DPQQIQQIFNQY348 (Figure S10, Table S2).

Sequence coverage across all samples ranged from 96% to 100%, enabling us to detect four mutations and differentiate between the K12 and BL21 strains (Figure S10). Table S2 summarizes the mutations detected in each sample, verification of the N- and C-terminal ends, and overall sequence coverage. In contrast, Table S3 summarizes the number of PSMs associated with each mutation, which allows us to confirm their relative abundance (Table S3). Leuginase shows a higher relative abundance of BL21-specific mutations, with A49 at 13%, N86 at 53%, S274 at 28%, and N285 detected as 1 PSM. In the pellet samples (P1–P3), we observed A49 at 3–8%, D86 at 51–63%, T274 at 18–26%, and N285 as 1 PSM, while similar results were observed in the broth samples (A49: 20–29%, D86: 45–52%, T274: 20–22%, N285: 1 PSM). The result for position 86 is difficult to confirm because deamidation (N86 → D), potentially induced by storage conditions, can confound the analysis. These findings (Table S4) suggest that the deamidation rate is lowest in Leuginase, intermediate in the pellet samples, and highest in the broth samples. Although position 285 was confirmed with only one PSM, the high-quality MS2 spectrum (Figure S11) validated the mutation. Overall, all four mutations (positions 49, 86, 274, and 285) were confirmed by MS2 spectra (Figure S11).

## Discussion

Many initiatives to produce EcA2 biosimilars or biobetters are underway, particularly due to the expiration of patents and the side effects of EcA2-based biopharmaceutical products currently in use. Additionally, some countries have started producing their own l-asparaginase formulations to minimize economic dependence and/or in response to frequent changes in global suppliers, which have occasionally led to shortages of specific l-asparaginase formulations.^35^ The limited availability of the reference product has also prompted low- and middle-income countries to begin manufacturing and supplying l-asparaginase biosimilars.

However, many l-asparaginase biopharmaceuticals are not therapeutically equivalent and may exhibit variations in the source of the active pharmaceutical ingredient, manufacturing processes, amino acid sequences, and/or composition of the final pharmaceutical product.^19–21,36–38^ These variations can affect the specific activity, substrate affinity, conformational stability, and immunogenicity.

One of the most critical and costly steps in biopharmaceutical production is purification as it must effectively remove cellular debris, lipopolysaccharides (LPS), DNA, and host cell proteins. The recombinant production protocols of the commercial products are not disclosed in sufficient detail; therefore, the scientific literature serves as the primary source of information on available protocols. There are several reports of recombinant production of EcA2 or its variants, where most focus on intracellular expression and others on secreted expression.^27,39^ None of these studies, however, compared the efficacy of their purification protocols side by side regarding the expression mode, either intracellular or secreted, particularly with respect to the quality of the final product.

EcA2 is administered primarily intravenously, and because of that, the purity and quality of this active pharmaceutical ingredient must be monitored following strict criteria. However, even after multiple purification steps, some contaminants may copurify with the therapeutic protein, potentially causing harm during patient treatment. In fact, significant amounts of impurities in commercial EcA2 products have been reported, and they may be associated with side effects, including hypersensitivity reactions in patients, which can impair the therapy.^34,40^

The present study focuses on the influence of the recombinant EcA2 source in *E. coli*, either intracellular or secreted, on the downstream process and the quality of the purified product. We performed three independent expressions of recombinant EcA2, producing it both intracellularly and secreted into the culture broth with both purified using the same protocol after preparation of the crude protein extract. We then analyzed the final products using technologies relevant to the critical quality attribute (CQA) requirements for the registration of new biopharmaceuticals, including the determination of the enzyme’s specific activity, verification of oligomers, characterization of conformational stability, determination of molecular integrity and identity, and identification of contaminants, including host cell proteins.

Differences in the chromatographic profiles during the protein capture step were observed with a higher amount of contaminant proteins detected in the soluble fraction of the cell pellet lysate. In the second chromatographic purification step, EcA2 from the soluble fraction of the cell pellet lysate showed a single peak corresponding to EcA2, whereas the chromatogram of the protein purified from the broth exhibited an additional peak close to EcA2, suggesting conformational heterogeneity in the enzyme purified from the broth or greater efficiency in separating contaminant proteins.

Regardless of the source, no statistically significant difference was detected in the specific activity of purified recombinant EcA2. Furthermore, both exhibited a similar three-dimensional structure and cooperative thermal denaturation profile with comparable thermal stability. However, the chemical identity analysis performed by ESI–MS revealed that the protein produced in the cell pellet presents a difference of 5 Da compared to that produced in the broth. We are investigating the reason for this difference, but it may be related to the oxidation or reaction of the protein with chemicals during expression and cell lysis.

Even though the purification steps for both proteins were the same, LC–MS/MS analyses revealed that the purification performed from the broth resulted in a lower number of HCP ions compared to the purification from the soluble fraction of the cell pellet lysate. This supports the idea that exporting recombinant proteins into the culture medium offers advantages over intracellular localization, including lower levels of protein contaminants, which reduces the number of steps required in downstream processing.^41^ The number and identity of HCP identified in EcA2 purified from the broth are similar to those found in the biopharmaceutical Leuginase.

We summarize the data related to the purity of the recombinant EcA2 produced from the broth or from the soluble fraction of the cell pellet lysate in Table 1. Additionally, we did not observe any significant differences in the 1D ^1^H NMR spectra of the samples nor did we detect any sharp signals indicative of small molecule contaminants.

**Table 1:** Summary of Data on Activity and Purity of Purified EcA2 Samples

protein source	yield (mg/L)^a^	specific activity (UI/mg)^b^	total activity (UI/mL)	HCP^c^	chemical modifications^d^
secreted	66.3 ± 2.3	136.3 ± 13.3	9.0 ± 0.9	56 ± 12 (111)	20 ± 2 (59)
intracellular	29.2 ± 4.6	123.5 ± 10.3	3.6 ± 0.6	194 ± 60 (294)	19 ± 3 (57)

a Mean and standard deviation from three independent recombinant productions.

b Mean and standard deviation from triplicates of three independent recombinant productions.

c Number of host cell proteins identified by LC–MS/MS analysis. The values represent the mean with standard deviation and the total protein hits observed in the three independent recombinant productions within parentheses.

d Chemical modifications include methionine oxidation and asparagine or glutamine deamidation. The values represent the mean with standard deviation and the total amounts of modifications observed in the three independent recombinant productions. Modified amino acids are indicated in Figure S7.

Recombinant biopharmaceuticals are typically produced from host cells, such as *E. coli*, yeast, and mammalian cells. Host cell proteins (HCPs) are impurities derived from the cellular strains used in the production process, which have the potential to be immunogenic and to inactivate the biopharmaceutical by proteolysis. Because they directly impact the safety and efficacy of biotherapeutic products, the overall quantity of residual HCPs is considered a critical quality attribute that must be analyzed, characterized, and controlled throughout the production process.^42,43^ The purification processes for therapeutic proteins must not only efficiently reduce these contaminants to acceptable levels in the final biopharmaceutical formulation but also demonstrate consistent reproducibility.

The enzyme-linked immunosorbent assay (ELISA) is commonly used to detect HCPs due to its high sensitivity and high-throughput capacity. However, a limitation of this assay is its lack of coverage and specificity in identifying and quantifying individual HCPs. To address this limitation, the LC–MS/MS has emerged as an orthogonal approach for identifying and providing semiquantitative data on individual HCPs at different stages of production and in the final product.^44,45^ Knowing the identities of each HCP is crucial for removing protein contaminants that are difficult to remove, ensuring the safety and efficacy of biotherapeutic products.^46–48^

*E. coli* l-asparaginase type 2 enzyme (ASPG2_ECOLI) was the most abundant protein, with the highest number of spectral counts of peptides among all analyzed proteins, and the sequence coverage obtained in all samples ranged from 94% to 98% (Table S1, Figure 7C). When analyzing the five most abundant HCPs based on spectral counting (Figure 7F), we identified at least two common proteins between pellet and supernatant purifications, such as glyceraldehyde-3-phosphate dehydrogenase A (G3P1_ECOLI) and transaldolase B (TALB_ECOLI). The glyceraldehyde-3-phosphate dehydrogenase A is a relatively abundant contaminant present in all of the purified EcA2 samples. This enzyme is composed of four identical 37 kDa subunits and is constitutively expressed in the cytoplasm^49^ and can be removed by affinity chromatography using a blue Sepharose CL-6B matrix.^50^ For Leuginase, the periplasmic oligopeptide-binding protein (OPPA_ECOLI) was the most abundant HCP. The HCP profile of the five most abundant proteins identified in our study demonstrates differences in the purifications of Leuginase and the proteins produced in the broth and cell pellet: the proteins identified in Leuginase originate from the periplasmic space and outer membrane, while the proteins identified from the cell pellet and broth originate from the cytosol and outer membrane (Figure 7F). This indicates that there is cell lysis during the expression or processing of the culture to separate the cells and debris from the broth supernatant.

In addition to the higher purity of the EcA2 samples isolated from the broth, our results indicate that purifying l-asparaginase from the cells appears to be less reproducible than that from the broth. This conclusion is based on the variability (standard deviation) in the recombinant EcA2 yield and the number of HCPs, as shown in Table 1. The purification of intracellular products requires cell lysis for recovery, which may increase the likelihood of coextracting cell wall components, including lipopolysaccharide (LPS)—also referred to as endotoxin, the major constituent of the outer membrane of Gram-negative bacteria.^51^ In contrast, the purification of products secreted into the culture medium can be performed without disrupting cellular integrity, thereby potentially reducing the level of LPS contamination and simplifying the downstream process. This is particularly relevant for recombinant products used in mammals, such as biopharmaceuticals, since LPS can elicit a strong pyrogenic response and, in severe cases, trigger septic shock.^52^ Therefore, although this study did not measure LPS levels, their quantification remains a critical step to rigorously evaluate biopharmaceutical quality control, as the chosen production method can directly impact endotoxin levels in the final product.

The complex production processes of biopharmaceuticals can cause unwanted chemical modifications to their amino acids, which can be detrimental to their stability and activity.^53^ All purified EcA2 samples exhibited chemical modifications, including methionine oxidation and deamidation of the Q or N side chains. However, the extent of these modifications was similar across all samples, indicating that the source of EcA2 did not influence the chemical modifications found. Nevertheless, this chemical modification analysis needs to be performed on samples purified from cultures grown in a bioreactor using larger broth volumes and longer cultivation times, which might increase the number of modifications in the purified EcA2.

It is worth noting that while many studies describe the purification of EcA2 secreted from *E. coli*, we did not find any work that directly compares these methods with purification from intracellular sources, leaving a gap for making an informed protocol choice. Reported yields range from 10 to 5240 mg/L of EcA2, with specific activities between 91 and 190 UI/mg, values that align well with our results.^24,25,54,55^ Further optimization of the protocols described here, particularly by conducting them after recombinant EcA2 expression in a bioreactor with larger volumes, is essential for achieving a robust technoeconomic analysis of both processes.

## Materials and Methods

### Reagents and Materials

Tryptone, yeast extract, and agar for microbiological cell culture were from KASVI (cat. no. K25-1612, 1702, and 1800; Curitiba, Brazil). Sodium phosphate monobasic, sodium phosphate dibasic, sodium chloride, potassium phosphate monobasic, potassium phosphate dibasic, ammonium sulfate, isopropyl β-d-1-thiogalactopyranoside (IPTG), and sodium ampicillin were from Sigma-Aldrich (St. Louis, MO, USA). The HiTrap Q HP column and Phenyl Sepharose CL-4B resin were from Cytiva (Marlborough, MA, USA). Amicon concentrators (30 kDa) were from Millipore (Billerica, MA, USA).

### Recombinant l-Asparaginase Expression

The coding sequence of *E. coli* type II l-asparaginase (GenBank: CAQ33267.1), comprising amino acids 23 to 348 optimized for *E. coli* expression containing the pelB leader peptide at the N-terminus, was cloned into plasmid pET25b using *Bam*HI and NdeI restriction sites by GenScript USA Inc. (Piscataway, NJ, USA). The construct has a theoretical average mass of 34593.90 and a monoisotopic mass of 34572.53 (Expasy PeptideMass). Plasmid bearing the construct was transformed into *E. coli* BL21­(DE3) (Sigma-Aldrich, St. Louis, MO, USA). The cells were cultured for 18 h in 80 mL of Luria–Bertani broth (LB) or in 40 mL of terrific broth (TB), both supplemented with 1% glucose and 50 μg/mL ampicillin, with agitation at 200 rpm and 30 °C. To induce intracellular production, the LB culture was transferred to three 2.5 L Ultra Yield flasks (Carlsbad, CA, USA), each containing 475 mL of LB with ampicillin (50 μg/mL), and incubated with shaking at 200 rpm and 37 °C for 4 h. When the cultures reached an OD_600nm_ of 1.0, IPTG was added to a final concentration of 0.2 mM, and the cells were incubated for 4 h at 37 °C with agitation at 200 rpm. To induce extracellular expression, the TB culture was transferred to three 2.5 L Ultra Yield flasks (Carlsbad, CA, USA), each containing 237.5 mL of TB with ampicillin (50 μg/mL), and incubated with shaking at 200 rpm and 37 °C for approximately 4 h until the culture reached an OD_600nm_ of 5.0. IPTG was added to a final concentration of 0.2 mM, and the cells were incubated for 20 h at 37 °C with agitation at 200 rpm.

### Protein Capture from Broth

Cells were separated by centrifugation at 8,000*g* for 30 min. The supernatant was vacuum-filtered sequentially through 0.45 and 0.22 μm membranes. The cleared supernatant (250 mL) was concentrated by ultrafiltration using a Pall Minimate tangential flow filtration system (Pall Corporation, USA) with a 30 kDa membrane (Pellicon XL Cassette, Merck Millipore, Germany) to a final volume of 100 mL. This sample was diafiltered with three volumes of buffer B (25 mM sodium phosphate, pH 7.4) at 50 mL/min and then concentrated to 80 mL. Solid (NH_4_)_2_SO_4_ was slowly added to the sample under stirring in an ice bath at a rate of 1 g/min until reaching a final concentration of 1 M in a total volume of 100 mL (adjusted at the end of this step). This sample was loaded onto a Phenyl Sepharose CL-4B column (50 × 50 mm) at a flow rate of 4 mL/min and equilibrated with buffer A (25 mM sodium phosphate, 1 M (NH_4_)_2_SO_4_, pH 7.4). The column was washed with buffer A until the A_280nm_ reached a steady value of about 0.04. Bound proteins were eluted using a linear gradient of buffer B from 0 to 60% over 150 mL, followed by 100% buffer B for 120 mL. The eluted samples were fractionated and analyzed on a 15% SDS-PAGE stained with colloidal Coomassie Blue G-250.

### Protein Capture from Cells Grown in LB

Cells were harvested by centrifugation at 8000*g* for 30 min and resuspended in 100 mL of buffer B with a SigmaFast protease inhibitor. Cells were disrupted by intermittent sonication at 12–16 Ω for 30 min, with 5 s on and 5 s off cycles (Microson Ultrasonic Cell Disruptor, Qsonica, LLC, Newtown, CT), in an ice bath for 1 h. The homogenate was centrifuged at 8000*g* for 30 min at 6 °C. The supernatant was filtered through a 0.22 μm membrane and then loaded onto a Phenyl Sepharose CL-4B column, as described above.

### Protein Polishing by Anion Exchange Chromatography

Protein fractions from hydrophobic interaction chromatography were diafiltered with 25 mM sodium phosphate buffer (pH 7.4) until reaching a conductivity of 4.0 mS/cm, using a Pall Minimate tangential flow filtration system (Pall Corporation, USA) with a 30 kDa membrane (Pellicon XL Cassette, Merck Millipore, Germany). The sample was concentrated to 30 mL at a flow rate of 50 mL/min and a pressure of 1.5–2.0 bar. This sample was then loaded onto a 5 mL HiTrap Q FF column (Cytiva, USA), equilibrated with buffer B. The sample was injected at a flow rate of 1 mL/min, and the proteins were eluted from the column with a 0–30% gradient of buffer C (25 mM sodium phosphate buffer with 1 M sodium chloride, pH 7.4) at a flow rate of 2 mL/min. After 25 min of gradient elution, the buffer C concentration was increased to 100% to elute the contaminants. The eluted samples were fractionated and analyzed on a 15% SDS-PAGE stained with colloidal Coomassie Blue G-250.

### Enzymatic Activity

The activity of the recombinant enzyme l-asparaginase 2 was evaluated by monitoring l-asparagine absorption at 225 nm, using a Jasco V-730 spectrophotometer (Jasco, Japan). This procedure is an adaptation of the original protocol described previously.^17^ The assay is conducted in a quartz cuvette with a 10 mm optical path, containing 100 mM sodium phosphate (pH 8.0), 20 mM l-asparagine, and 50 μg/mL l-asparaginase 2. The reaction was monitored for 10 min, with an integration time of 2 s, at 37 °C.

### One-Dimensional Proton Nuclear Magnetic Resonance (1D ^1^H NMR)

Samples collected after the final purification step were supplemented with 10% D_2_O and 1 mM sodium 2,2-dimethyl-2-silapentane-5-sulfonate (DSS) and then analyzed by 1D ^1^H NMR at 25 °C on a 400 MHz Bruker Avance III spectrometer equipped with a broadband inverse *Z*-axis gradient probe. Spectra were processed and analyzed using the TopSpin 4.0.6 software (Bruker Spin Corp., GmbH).

### Intrinsic Fluorescence Spectroscopy

Intrinsic fluorescence assays were performed using a Jasco FP-8250 spectrofluorometer (Jasco, Japan) with a quartz cuvette having a 5 mm optical path length. Fluorescence spectra were obtained using excitation set at 295 nm and emission recorded from 300 to 400 nm using 20 μM of the produced recombinant enzymes diluted in 10 mM sodium phosphate (pH 7.4), with a spectral resolution of 0.5 nm, a scanning speed of 100 nm/min, and two accumulations. Thermal denaturation was monitored at 340 nm while the samples were heated from 25 to 80 °C in 0.5 °C increments at a rate of 1 °C/min. The data were analyzed using GraphPad Prism 6 (GraphPad Software, USA).

### Size Exclusion Chromatography

The hydrodynamic radius of the produced recombinant enzymes was estimated using an analytical size exclusion chromatography column (TSKgel G3000SWXL, 300 × 7.8 mm, Supelco), with 10 μM recombinant enzymes diluted in 10 mM sodium phosphate (pH 7.4). Proteins were separated at a flow rate of 1 mL/min and monitored at 280 nm. Fractions from each peak were collected and analyzed by 15% SDS-PAGE, and the samples were stained with colloidal Coomassie Blue G-250. The following globular proteins were used to construct a standard curve of elution profile versus hydrodynamic radius: thyroglobulin (86 Å, Sigma-Aldrich, cat. no. T9145), bovine serum albumin dimer (45.6 Å), and monomer (36.2 Å, Sigma-Aldrich, cat no. A7906).

### Characterization by Electrospray Ionization (ESI)–Mass Spectrometry (ESI–MS) Analysis

The intact mass of the asparaginase (50 μM in 0.1% formic acid in water/acetonitrile 50:50) was measured in a Traveling Wave Ion Mobility Mass Spectrometer (TWIM-MS, Synapt G1 HDMS, Waters, UK) in positive mode with a capillary voltage of 3.0 kV by direct infusion at 20 μL/min for 10 min, with scan time 3 s, interscan time 0.02 s, sampling cone 40, extraction cone 4.0, collision energy 6.0 (trap) and 4.0 (transfer), N_2_ used as mobility gas at 0.4 bar, source temperature 80 °C, gas flow 5.0 mL/min, IMS gas flow 40.0 mL/min, desolvation temperature of 250 °C, and flow of 500 L/h. Measurements were performed between *m*/*z* 500 and 3000. Calibration was performed with 0.1% phosphoric acid (v/v) in acetonitrile/H_2_O (1:1). Other typical instrumental settings followed a previous study.^56^ Data were analyzed with ProteinLynx (Waters Corporation, UK).^57^ All measurements were performed at the Laboratory of Proteomics and Mass Spectrometry (UEMP-IBqM-UFRJ), Rio de Janeiro, RJ, Brazil.

### Liquid Chromatography–Mass Spectrometry (LC–MS/MS) Analysis of Host Cell Proteins

Total protein quantification was performed by using Qubit 2.0 (Invitrogen). An aliquot of 50 μg of protein was reduced with 10 mM dithiothreitol in the presence of 2 M urea, 0.5 M thiourea, and 0.1 M triethylammonium bicarbonate buffer (pH 8.5) at 37 °C for 1 h. The samples were alkylated with 25 mM iodoacetamide at 25 °C for 20 min in the dark. In-solution digestion with trypsin (1:50, enzyme/substrate ratio) was performed in the presence of 1.2 M urea, 0.3 M thiourea, and 0.1 M triethylammonium bicarbonate buffer (pH 8.5) at 37 °C for 16 h. The reaction was quenched with 1% trifluoroacetic acid (TFA) and desalted using StageTip C18 (one disk of 3 M Empore +1 mg Poros R2 resin) with minor modifications.^58^ Tryptic peptides eluted in 70% acetonitrile containing 0.1% TFA were dried in a SpeedVac and stored at −20 °C until further analysis. Samples were reconstituted in 30 μL of 0.1% formic acid and quantified using the Qubit 2.0 fluorometer (Invitrogen).

Tryptic peptides (2.0 μL, 0.5 μg/μL) were analyzed on an Orbitrap Exploris 480 mass spectrometer (Thermo Scientific) coupled with a Vanquish Neo system (Thermo Scientific) for LC–MS/MS. Peptides were trapped on a PepMap Neo C18 precolumn (5 × 0.3 mm i.d., 5 μm particles, Thermo Scientific) and separated using a PepMap RSLC C18 column (750 × 0.075 mm i.d., 2 μm particle, 100 Å, Thermo Scientific) heated at 60 °C. A 60 min gradient was applied with solvent A (0.1% formic acid in water) and solvent B (0.1% formic acid, 80% acetonitrile in water) as follows: 2–50% solvent B (0–50 min), 50–99% solvent B in 2.5 min, and 99% solvent B for 7.5 min, at a flow rate of 300 nL/min. The mass spectrometer operated in data-dependent acquisition (DDA) mode with 20 scans and an EASY-IC at the start. Full-scan MS was acquired in the range of *m*/*z* 375–1500 with a resolution of 120,000 (fwhm) at *m*/*z* 200, 50% of RF lens, 300% normalized AGC, automatic ion time (IT), and 1 microscan. The intensity threshold for the MS2 event was set to 8000 counts. Fragment ions were acquired at 15,000 resolution (fwhm) at *m*/*z* 200, with a 2 *m*/*z* isolation window, 30% normalized HCD, 50% normalized AGC, automatic IT, and 1 microscan.

Raw files were converted to mzML using MSconvert v3.0.22133,^59^ uploaded to *FragPipe* v22.0,^60^ and the MS2 spectra were searched using the MSFragger v4.1 engine against the *E. coli* strain BL21­(DE3) FASTA file (UP000002032_469008; January 2025; 4156 entries) including the common contaminants.^61^ Precursor and fragment ion mass tolerances were set to 10 ppm and 0.02 Da, respectively. Carbamidomethyl cysteine was set as a fixed modification, while methionine oxidation and protein N-terminal acetylation were set as variable modifications. Semitrypsin digestion with a maximum of 3 missed cleavages and 3 variable modifications per peptide was selected. Peptide and protein identifications were filtered at 1% FDR using Philosopher v5.1.1.^62^ An open search with the default parameters using *PTM-Shepperd* was performed.^63^ A second analysis in FragPipe was performed using the same FASTA but modified with the sequences of EcA2 of strain BL21 (GenBank: CAQ33267.1) and K12 (GenBank: GCA_009832885.1), with 326 amino acids (L23-Y348) and the N-terminus with or without pelB peptide (1MKYLLPTAAAGLLLLAAQPAMA22, Q04085_PECCA) and including some PTMs detected in the open search. The *FragPipe-PDV* viewer^64^ was used to visualize the protein coverage and the MS2 spectra. Downstream analysis was carried out using Perseus v2.0.11,^65^ removing common contaminants, and identified proteins were filtered by ≥2 unique peptides and ≥2 unique spectral counts. Gene Ontology annotations, subcellular location, and molecular weight were downloaded from UniProt (January 2025) and imported to Perseus. Venn diagrams and upset plots were plotted in R v4.4.2 using the ggVennDiagram v1.5.2, while ggplot2 v3.5.1 was used for the bubble, bar, and dot plots. Isoelectric points were downloaded using the Expasy tool “Compute pI/Mw” (https://web.expasy.org/compute_pi/).

## Supplementary Material





## Notes

Extended and raw data on all analyses reported in the main figures (PDF)
Total spectral count (XLSX)
