# Supplementary material for: Investigation of the Impact of a Protein Source on the Purification of l‑Asparaginase Type II from Escherichia coli
Source: ACS Omega. 2025 Jun 13;10(27):29166–79. doi: 10.1021/acsomega.5c01816 (PMC12273519; doi:10.1021/acsomega.5c01816)
Supplement: Supplementary file 1 [file ao5c01816_si_001.pdf]

Supporting Information

Investigation of the Impact of Protein Source on the Purification of L-Asparaginase Type II from *Escherichia coli*

Anna Catharinna da Costa[1,2#]; Talita Stelling de Araujo[1#]; Anna Carolina Lomba Pereira[1]; Luis Ariel Espinosa Rodríguez[3]; Leonardo Dingo do Lago[1]; Camila Dias Leite da Silva[1]; Rafael Alves de Andrade[1]; Luís Maurício Trambaioli da Rocha e Lima[2,4,5*]; Fábio C. S. Nogueira[3*]; Gilberto Barbosa Domont[3*]; Marcius da Silva Almeida[1,2*]

[1] Protein Advanced Biochemistry (PAB), Institute of Medical Biochemistry (IBqM) -National Center for Structural Biology and Bioimaging (CENABIO), Universidade Federal do Rio de Janeiro, Rio de Janeiro, RJ, Brazil.

[2] Programa de Pós-Graduação em Química Biológica, Universidade Federal do Rio de Janeiro, Rio de Janeiro, RJ, 21941-902, Brazil.

[3] Centro de Pesquisa em Medicina de Precisão (CPMP), Universidade Federal do Rio de Janeiro, Rio de Janeiro, RJ 21941-902, Brazil.

[4] Laboratório de Biotecnologia Farmacêutica (pbiotech), Faculdade de Farmácia, Universidade Federal do Rio de Janeiro, Rio de Janeiro, RJ 21941-902, Brazil.

[5] Programa de Pós-Graduação em Ciências Farmacêuticas, Faculdade de Farmácia, Universidade Federal do Rio de Janeiro, Rio de Janeiro, RJ, 21941-902, Brazil.

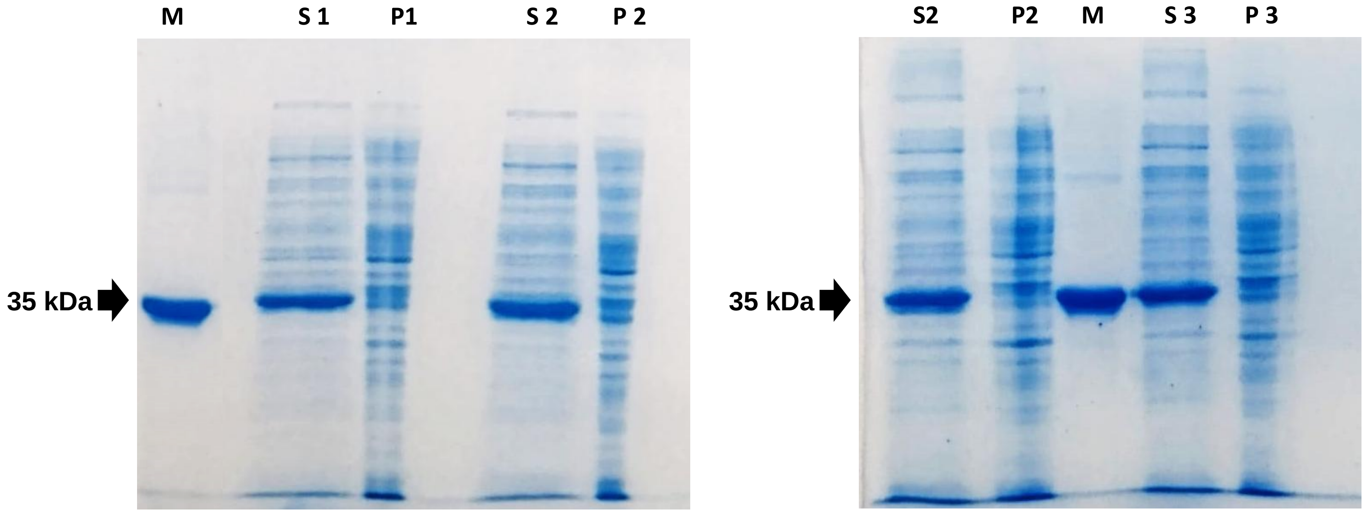

Fig S1. Uncropped gel of the expression of L-Asparaginase type 2 from *E. coli* BL21 (DE3) broth. 15% SDS-PAGE gels stained with colloidal Coomassie Blue. A. Lanes: M, EcA2 used as molecular mass standard (35 kDa); T1-3, samples from cultures 1, 2 and 3 (pellet + supernatant) at the end of induction; S1-3, samples of the supernatant of cultures 1, 2 and 3 at the end of induction; P1-3, samples of the cell pellet from cultures 1, 2 and 3 at the end of induction.

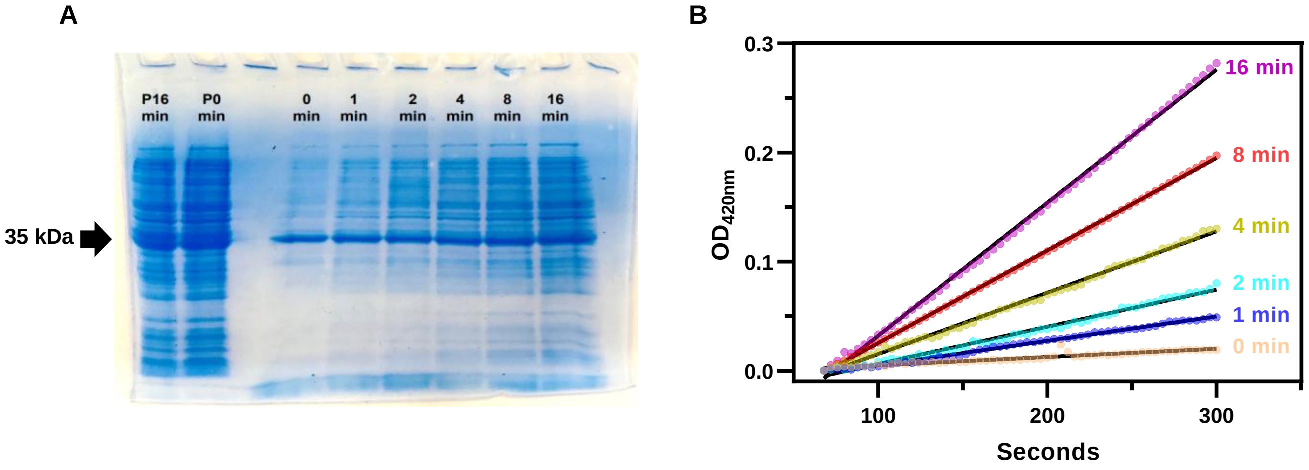

Fig S2. Influence of cell lysis on the protein content in the broth. **A**. PAGE-SDS 15% stained by colloidal Coomassie Blue. Lane P0, cell pellet before lysis; Lane P16, cell pellet after 16 min of lysis by sonication; Lanes 0-16, broth before and after cell lysis by sonication for 1, 2, 4, 8, and 16 minutes. **B**. β-galactosidase activity in broth before and after cell lysis by sonication.

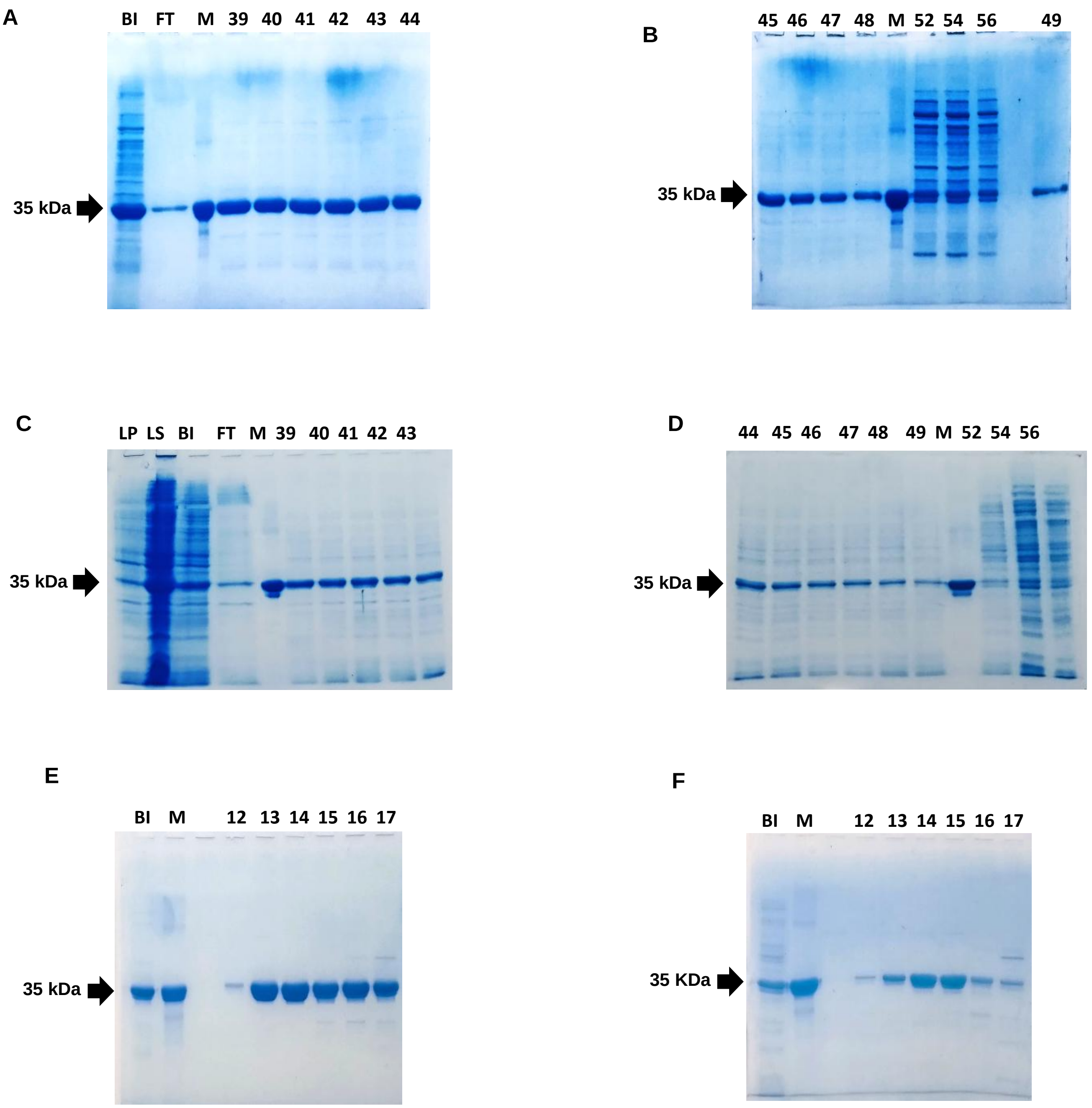

Fig S3. Uncropped gels of hydrophobic interaction (A-D) and anion exchange (E-F) chromatographies for purifying L-asparaginase type 2 from the broth (A, B, E) and the soluble fraction of the cell pellet lysate (C, D, F). Fractions from each chromatography were evaluated by 15% SDS-PAGE stained with colloidal Coomassie Blue. SDS-PAGE in panels A, B and E refer to the broth purification (Fig 3A), in C, D and F, to the soluble fraction of the cell pellet lysate purification. BI, sample before injecting in the column; FT, flow through; M, EcA2 standard with 35 kDa; LP, lysed pellet; LS, lysis supernatant; F39-56, the protein fractions collected during the hydrophobic interaction chromatography; F12-17, the protein fractions collected during the anion exchange chromatography.

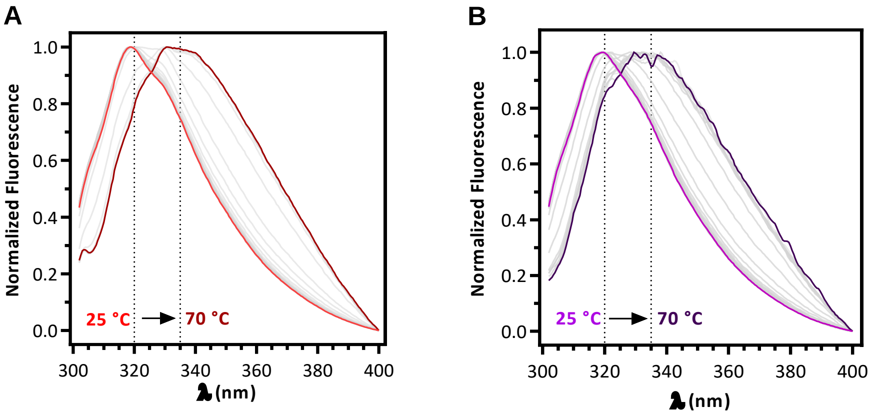

Fig S4. Intrinsic fluorescence spectra from thermal denaturation analysis of L-asparaginase produced in the broth (A) and the cell pellet lysate (B). Vertical lines indicate the maximum fluorescence emission wavelengths of the variants at 25 °C (319 nm) and 70 °C (335 nm). Intrinsic fluorescence spectra were collected with excitation at 295 nm and emission ranging from 300 nm to 400 nm.

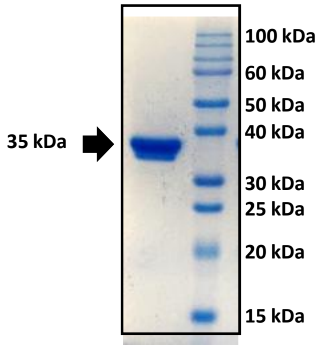

Fig S5. Molecular weight standard. Lane 1, *E. coli* L-asparaginase type 2 (EcA2); Lane 2, PageRuler Unstained Protein Ladder (ThermoFisher).

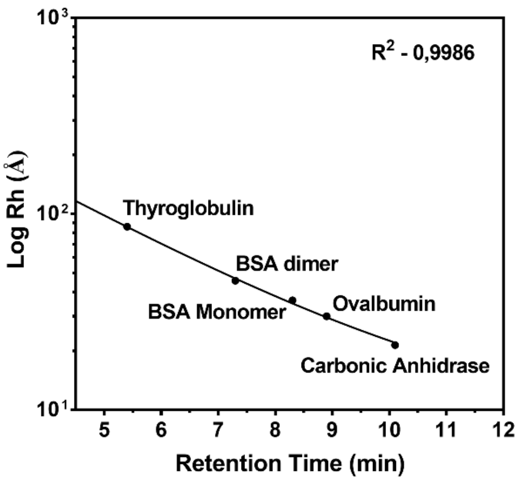

Fig S6. TSK-Gel G2000SW$_{XL}$ column standard curve. The elution times were plotted against the hydrodynamic radius of each standard protein and fitted to an exponential one-phase decay function. The standard globular proteins used and their hydrodynamic radius were thyroglobulin – 86 Å, bovine serum albumin – dimer 45.6 Å and monomer 36.2 Å, ovalbumin– 30.5 Å, and carbonic anhydrase – 21.4 Å.

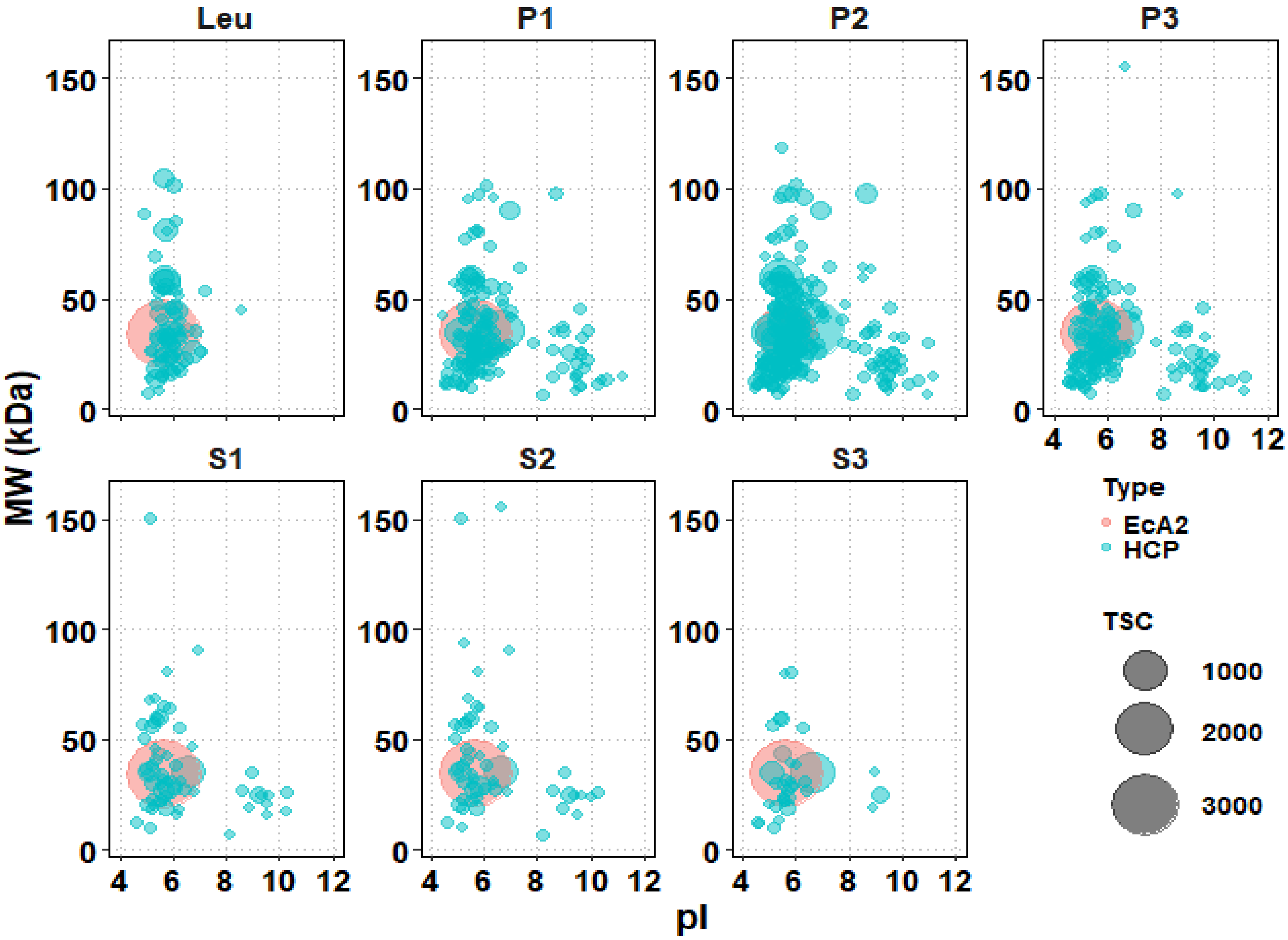

Fig S7. Bubble plots showing the Molecular Weight (MW) vs pI of L-Asparaginase II (EcA2) and HCPs in all samples. Bubble size corresponds to Total Spectral Counts (TSC). The MW of EcA2 is 36.8 kDa.

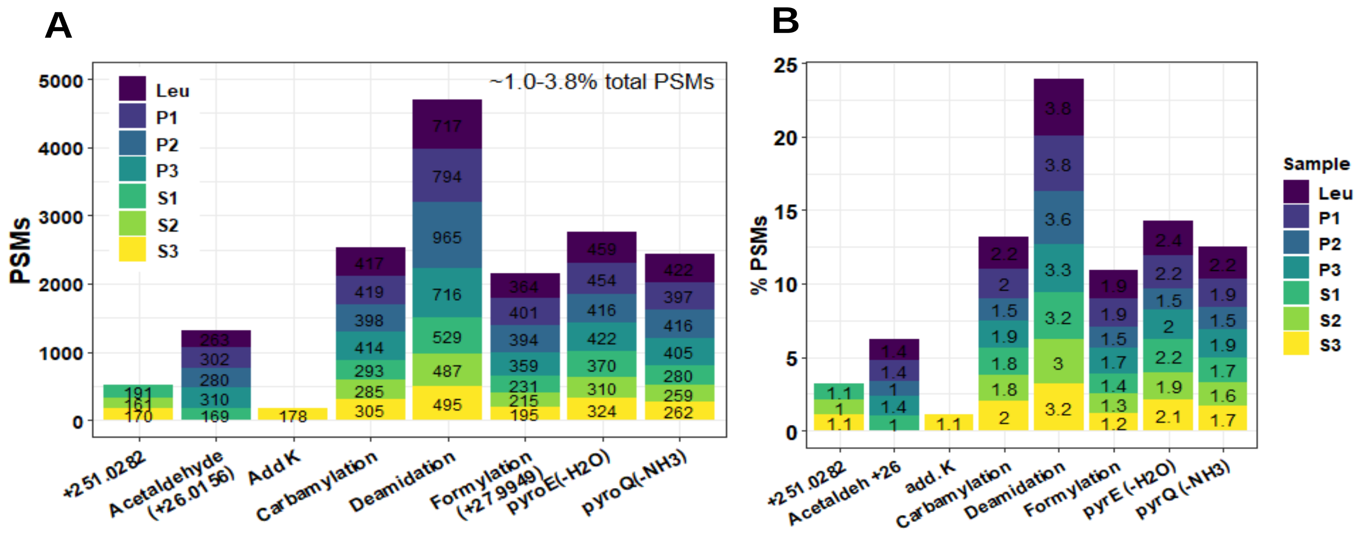

Fig S8. Bar plots of the PTM-Shepperd results of the most abundant modifications detected in each sample analyzed. (A) Number of PSMs and (B) % of PSMs identified in total.

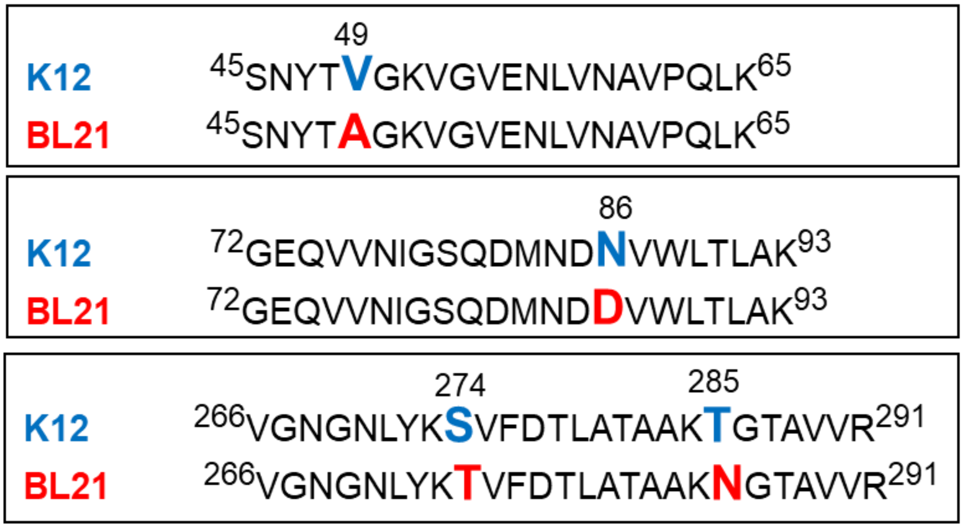

Fig S9. Amino acid mutations across the two EcA2 strains of K12 and BL21 (DE3) considering the signal peptide (1M-A22) + mature protein (23L-Y348).

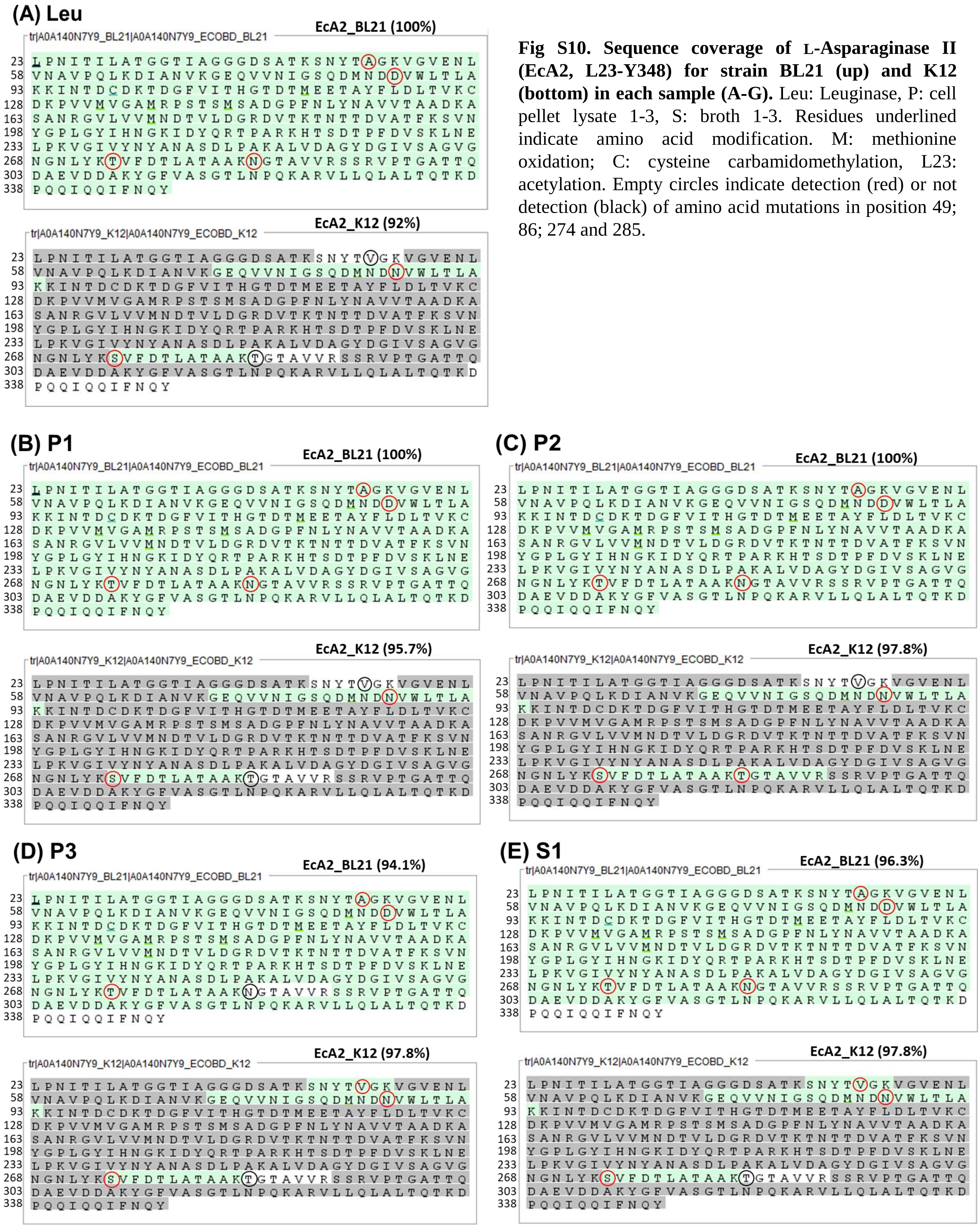

Fig S10. Sequence coverage of L-Asparaginase II (EcA2, L23-Y348) for strain BL21 (up) and K12 (bottom) in each sample (A-G). Leu: Leuginase, P: cell pellet lysate 1-3, S: broth 1-3. Residues underlined indicate amino acid modification. M: methionine oxidation; C: cysteine carbamidomethylation, L23: acetylation. Empty circles indicate detection (red) or not detection (black) of amino acid mutations in position 49; 86; 274 and 285.

(F) S2

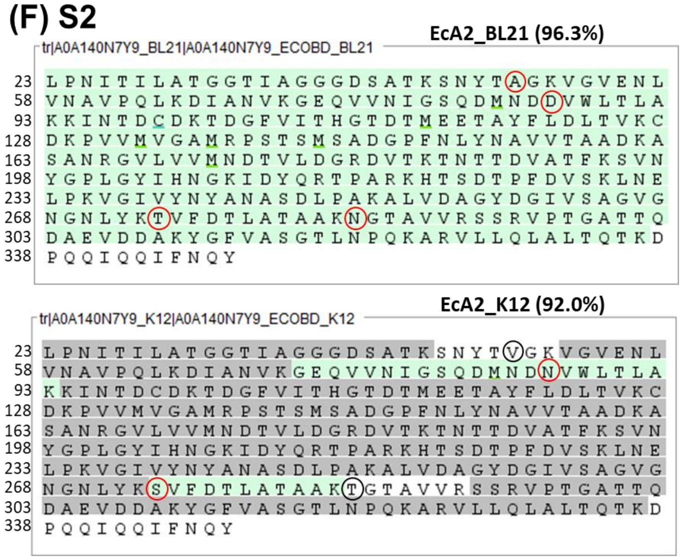

EcA2_BL21 (96.3%)
tr|A0A140N7Y9_BL21|A0A140N7Y9_ECOBD_BL21
23 LPNITILATGGTIAGGGDSATKSNYTAGKVGVENL
58 VNAVPQLKDIANVKGEQVVNIGSQDMNDDVWLTLA
93 KKINTDCDKTDGFVITHGTDTMEETAYFLDLTVKC
128 DKPVVMVGAMRPSTSMSADGPFNLYNAVVTAADKA
163 SANRGVLVVMNDTVLDGRDVTKTNTTDVATFKSVN
198 YGPLGYIHNGKIDYQRTPARKHTSDTPFDVSKLNE
233 LPKVGIVYNYANASDLPAKALVDAGYDGIVSAGVG
268 NGNLYKTVFDTLATAAKNGTAVVRSSRVPTGATTQ
303 DAEVDDAKYGFVASGTLNPQKARVLLQLALTQTKD
338 PQQIQQIFNQY
EcA2_K12 (92.0%)
tr|A0A140N7Y9_K12|A0A140N7Y9_ECOBD_K12
23 LPNITILATGGTIAGGGDSATKSNYTVGKVGVENL
58 VNAVPQLKDIANVKGEQVVNIGSQDMNDNVWLTLA
93 KKINTDCDKTDGFVITHGTDTMEETAYFLDLTVKC
128 DKPVVMVGAMRPSTSMSADGPFNLYNAVVTAADKA
163 SANRGVLVVMNDTVLDGRDVTKTNTTDVATFKSVN
198 YGPLGYIHNGKIDYQRTPARKHTSDTPFDVSKLNE
233 LPKVGIVYNYANASDLPAKALVDAGYDGIVSAGVG
268 NGNLYKSVFDTLATAAKTGTAVVRSSRVPTGATTQ
303 DAEVDDAKYGFVASGTLNPQKARVLLQLALTQTKD
338 PQQIQQIFNQY

(G) S3

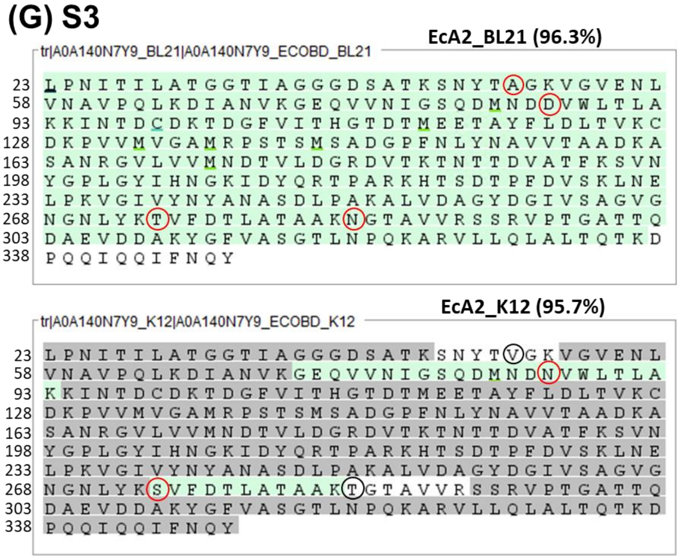

EcA2_BL21 (96.3%)
tr|A0A140N7Y9_BL21|A0A140N7Y9_ECOBD_BL21
23 LPNITILATGGTIAGGGDSATKSNYTAGKVGVENL
58 VNAVPQLKDIANVKGEQVVNIGSQDMNDDVWLTLA
93 KKINTDCDKTDGFVITHGTDTMEETAYFLDLTVKC
128 DKPVVMVGAMRPSTSMSADGPFNLYNAVVTAADKA
163 SANRGVLVVMNDTVLDGRDVTKTNTTDVATFKSVN
198 YGPLGYIHNGKIDYQRTPARKHTSDTPFDVSKLNE
233 LPKVGIVYNYANASDLPAKALVDAGYDGIVSAGVG
268 NGNLYKTVFDTLATAAKNGTAVVRSSRVPTGATTQ
303 DAEVDDAKYGFVASGTLNPQKARVLLQLALTQTKD
338 PQQIQQIFNQY
EcA2_K12 (95.7%)
tr|A0A140N7Y9_K12|A0A140N7Y9_ECOBD_K12
23 LPNITILATGGTIAGGGDSATKSNYTVGKVGVENL
58 VNAVPQLKDIANVKGEQVVNIGSQDMNDNVWLTLA
93 KKINTDCDKTDGFVITHGTDTMEETAYFLDLTVKC
128 DKPVVMVGAMRPSTSMSADGPFNLYNAVVTAADKA
163 SANRGVLVVMNDTVLDGRDVTKTNTTDVATFKSVN
198 YGPLGYIHNGKIDYQRTPARKHTSDTPFDVSKLNE
233 LPKVGIVYNYANASDLPAKALVDAGYDGIVSAGVG
268 NGNLYKSVFDTLATAAKTGTAVVRSSRVPTGATTQ
303 DAEVDDAKYGFVASGTLNPQKARVLLQLALTQTKD
338 PQQIQQIFNQY

(A)

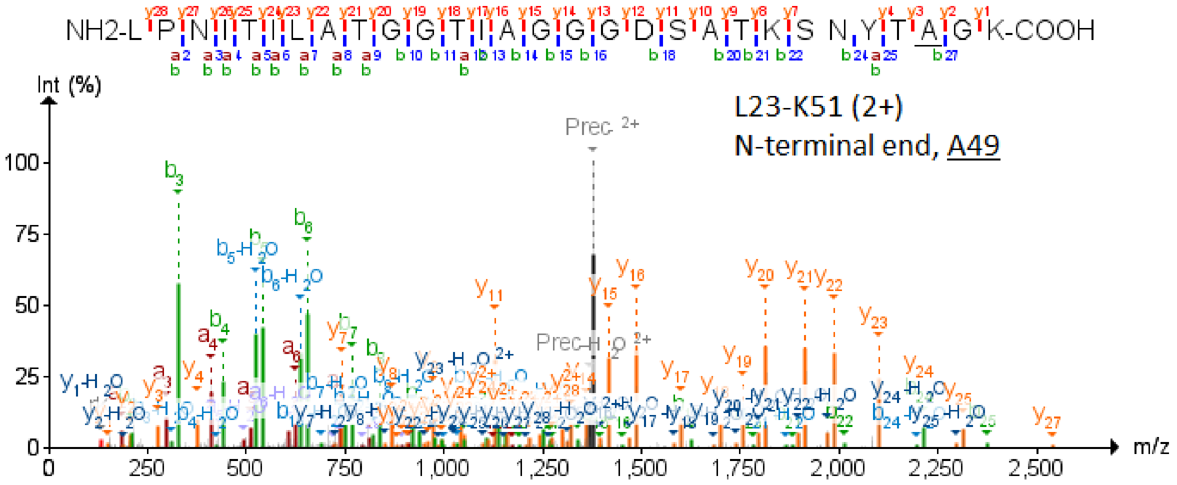
NH2-L P N I T I L A T G G T I A G G G D S A T K S N Y T A G K-COOH
Int (%)
L23-K51 (2+)
N-terminal end, A49
Prec 2+
m/z

(B)

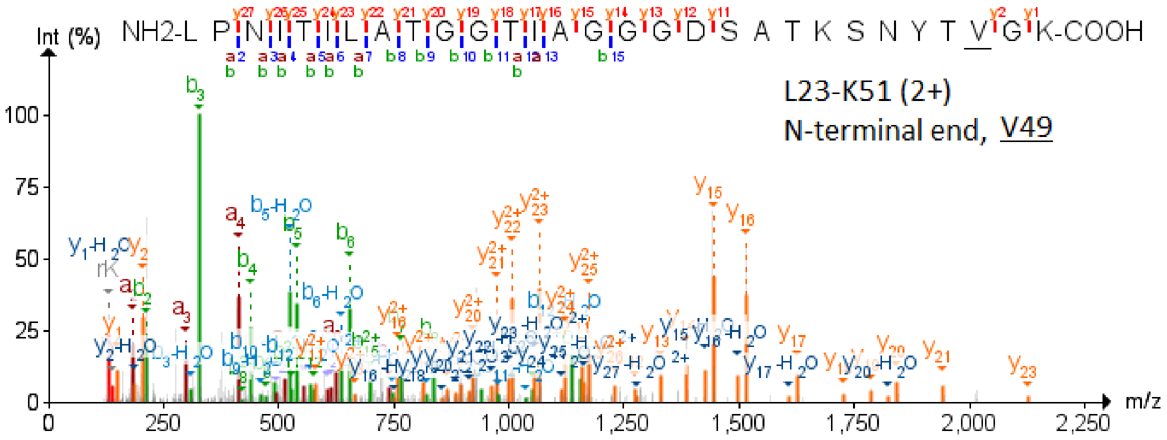
NH2-L P N I T I L A T G G T I A G G G D S A T K S N Y T V G K-COOH
Int (%)
L23-K51 (2+)
N-terminal end, V49
m/z

(C)

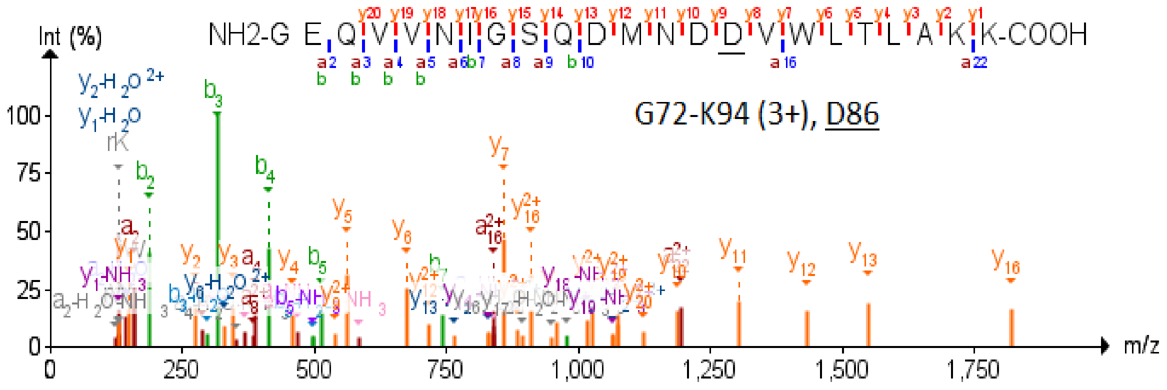
NH2-G E Q V V N I G S Q D M N D D V W L T L A K K-COOH
Int (%)
G72-K94 (3+), D86
m/z

(D)

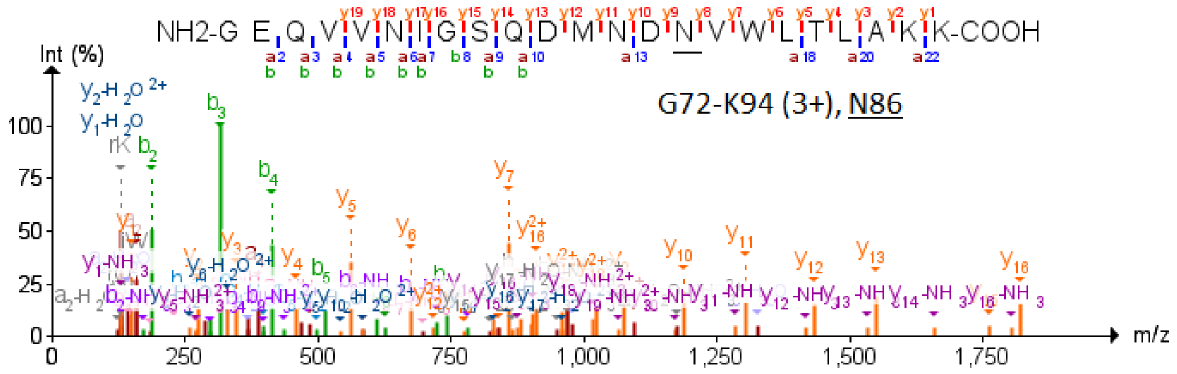

(E)

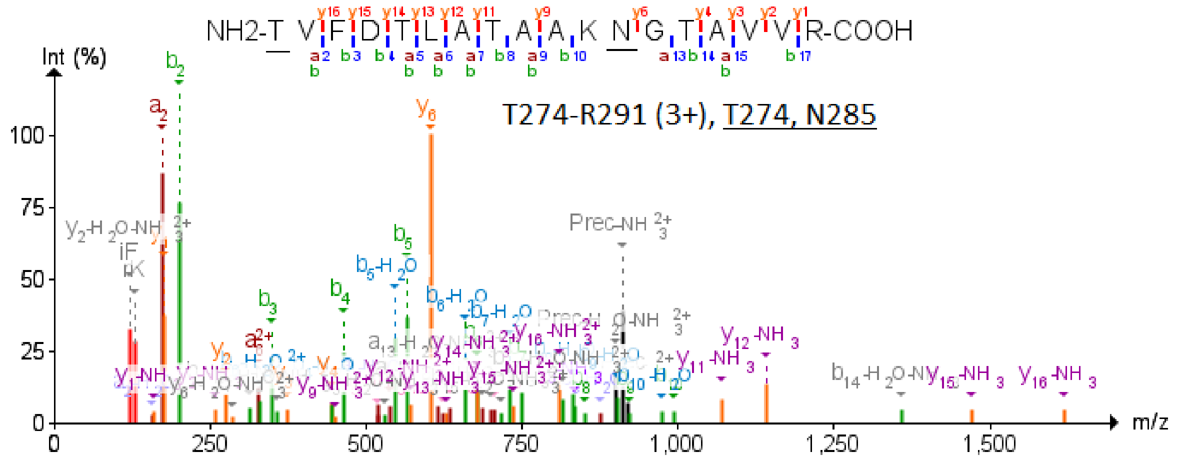

(F)

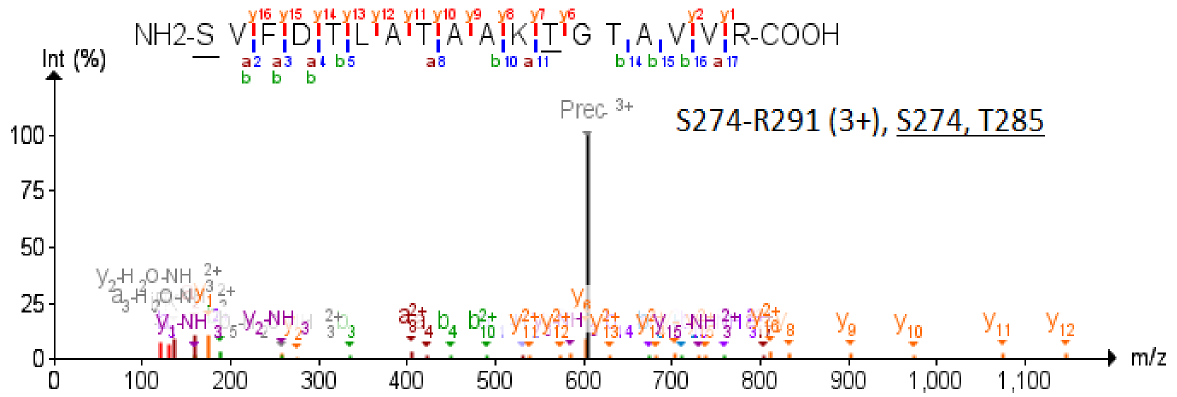

Fig S11. MS/MS spectra of peptides with detected mutations in amino acid position 49 (A-B), 86 (C-D), 274 and 285 (E-F), found in the biopharmaceutical Leuginase® and in the protein present in the broth and the cell pellet lysate samples. N-terminal end (L23-K51) of L-ASNase 2 (ASPG2_ECOLI) was detected in (A-B).

Table S2. Detected mutations, *N/C*-terminal ends, and sequence coverage of EcA2 by LC-MS/MS of proteolytic peptides.

Samples	Amino acid position [b]				*N/C*-terminus verified		EcA2 Total Coverage (%) [a]
	49	86	274	285	Nt	Ct	
Leu	A	D, N	T, S	N	L23	Y348	100
P1	A	D, N	T, S	N	L23	Y348	100
P2	A	D, N	T, S	N, T	L23	Y348	100
P3	A, V	D, N	T, S	n.d	L23	Y348	97.8
S1	A, V	D, N	T, S	N	L23	Y348	100
S2	A	D, N	T, S	N	L23	K326	96.3
S3	A	D, N	T, S	N	L23	Y348	100

(a) Total coverage of the mature protein (23L-Y348) and considering the identified peptides in both strains (BL21 and K12).
(b) n.d: Residue not-detected. Red and blue color correspond to mutations of EcA2 in BL21 and K12 strains, respectively.

Table S3. Number of PSMs in each of the four amino acid mutation of EcA2 detected by LC-MS/MS.

Samples	# of unique PSMs (Total / Intens > 0) for each site[a]								Total unique PSMs (BL21/ K12)
	A49	V49	D86	N86	T274	S274	N285	T285	
Leu	49/12	nd	13/6	195/56	4/4	101/21	1/1	nd	67/296
P1	44/13	nd	272/79	33/10	87/16	35/7	1/1	nd	404/68
P2	12/4	nd	164/54	23/12	84/16	33/13	nd	nd	260/56
P3	36/19	1/1	276/79	20/6	100/26	3/1	nd	nd	412/24
S1	108/24	1/1	271/78	23/7	111/20	3/2	1/1	nd	491/27
S2	150/35	nd	238/66	21/5	104/20	4/1	1/1	nd	493/25
S3	137/32	nd	219/43	14/6	110/20	5/5	1/1	nd	467/19

(a) Red and blue color correspond to mutations of EcA2 in BL21 and K12 strains, respectively. nd: not detected.

Table S4. Relative abundance (%) based on PSMs in each of the four amino acid mutation of EcA2 detected by LC-MS/MS.

Samples	Abundance (%, PSMs) of site mutations (BL21 & K12)[a]								Total unique PSMs
	A49	V49	D86	N86	T274	S274	N285	T285	
Leu	13,50		3,58	53,72	1,10	27,82	0,28		363
P1	9,32		57,63	6,99	18,43	7,42	0,21		472
P2	3,80		51,90	7,28	26,58	10,44	0,00		316
P3	8,26	0,23	63,30	4,59	22,94	0,69	0,00		436
S1	20,85	0,19	52,32	4,44	21,43	0,58	0,19		518
S2	28,96		45,95	4,05	20,08	0,77	0,19		518
S3	28,19		45,06	2,88	22,63	1,03	0,21		486

(a) Red and blue color correspond to mutations of EcA2 in BL21 and K12 strains, respectively
